# Inequalities in medicine use in Central Eastern Europe: an empirical investigation of socioeconomic determinants in eight countries

**DOI:** 10.1186/s12939-015-0261-0

**Published:** 2015-11-05

**Authors:** Sabine Vogler, August Österle, Susanne Mayer

**Affiliations:** Department of Health Economics, WHO Collaborating Centre for Pricing and Reimbursement Policies, Gesundheit Österreich GmbH (Austrian Public Health Institute), Vienna, Austria; Department of Socioeconomics, Institute for Social Policy, Vienna University of Economics and Business, Vienna, Austria; Department of Health Economics, Centre for Public Health, Medical University of Vienna, Kinderspitalgasse 15/1, 1090 Vienna, Austria

**Keywords:** Medicines, Access, Use, Inequalities, Education, Income, Affordability, Availability, Co-payments

## Abstract

**Background:**

Equitable access to essential medicines is a major challenge for policy-makers world-wide, including Central and Eastern European countries. Member States of the European Union situated in Central and Eastern Europe have publicly funded pharmaceutical reimbursement systems that should promote accessibility and affordability of, at least essential medicines. However, there is no knowledge whether socioeconomic inequalities exist in these countries. Against this backdrop, this study analyses whether socioeconomic determinants influence the use of prescribed and non-prescribed medicines in eight Central and Eastern European countries (Bulgaria, Czech Republic, Hungary, Latvia, Poland, Romania, Slovenia, Slovakia). Further, the study discusses observed (in)equalities in medicine use in the context of the pharmaceutical policy framework and the implementation in these countries.

**Methods:**

The study is based on cross-sectional data from the first wave of the European Health Interview Survey (2007–2009). Multivariate logistic regression analyses were carried out to determine the association between socioeconomic status (measured by employment status, education, income; controlled for age, gender, health status) and medicine use (prescribed and non-prescribed medicines). This was supplemented by a pharmaceutical policy analysis based on indicators in four policy dimensions (sustainable funding, affordability, availability and accessibility, and rational selection and use of medicines).

**Results:**

Overall, the analysis showed a gradient favouring individuals from higher socioeconomic groups in the consumption of non-prescribed medicines in the eight surveyed countries, and for prescribed medicines in three countries (Latvia, Poland, Romania). The pharmaceutical systems in the eight countries were, to varying degrees, characterized by a lack of (public) funding, thus resulting in high and growing shares of private financing (including co-payments for prescribed medicines), inefficiencies in the selection of medicines into reimbursement and limitations in medicines availability.

**Conclusion:**

Pharmaceutical policies aiming at reducing inequalities in medicine use require not only a consideration of the role of co-payments and other private expenditure but also adequate investment in medicines and transparent and clear processes regarding the inclusion of medicines into reimbursement.

**Electronic supplementary material:**

The online version of this article (doi:10.1186/s12939-015-0261-0) contains supplementary material, which is available to authorized users.

## Background

Ensuring equitable access to essential medicines is a major challenge for policy-makers. Essential medicines are those that satisfy the priority health care needs of the population and should be selected with due regard to disease prevalence, evidence on efficacy and safety, and comparative cost effectiveness. Essential medicines are intended to be available within the context of functioning health systems at all times, in adequate amounts, in the appropriate dosage forms, with assured quality, and at a price that the individual and the community can afford [1]. Countries have the responsibility to provide access to essential medicines while the implementation of the concept of essential medicines is intended to be flexible and adaptable to country specific situations [2].

The World Health Organization (WHO) has been promoting the concept of universal health coverage, arguing that timely access to health services, including medication, requires a well-functioning health financing system [3]. Related to pharmaceuticals, this means that, independently from the ownership of the suppliers (private or public suppliers and dispensaries), those medicines considered as essential in the national context are, at least partially, funded by the state, through a social health insurance system or a national health service.

Such reimbursement systems are in place in all Member States of the European Union (EU) where funding and reimbursement of medicines remains a national competence even if other areas (marketing authorization) have been harmonized [4]. This is also the case of the Central and Eastern European countries (CEECs) that are members of the EU. As of 2015, this is a total of eleven countries: Czech Republic, Estonia, Hungary, Latvia, Lithuania, Poland, Slovenia and Slovakia (acceding to the EU in 2004); Bulgaria and Romania (joining the EU in 2007); and Croatia which has been an EU Member State since mid-2013.

From the 1990s, the CEECs have changed from highly centralised and hospital-centred Semaskho systems towards social insurance systems with varying degrees of state centeredness and liberal market elements [5, 6]. In terms of pharmaceutical policies, the CEECs departed from the former principle of free medication for the entire population, starting to differentiate between medicines that should either be fully or partially reimbursed by the health system and introducing co-payment schemes for out-patient medicines [7–10].

In the new millennium, all these countries had positive lists, i.e. lists of medicines that may be prescribed at the expense of a third party payer (corresponding to the essential medicines lists concept). However, there are indications that the lists of (co-)funded medicines in the CEECs were smaller compared to those in the Western, Northern and Southern EU Member States [9, 10]. Further, medicines included in the positive lists were not necessarily 100 % funded since medicines whose therapeutic benefit was assessed to be lower were funded partially, with the remainder to be paid by out-patients in out-patient care [8–10]. In order to ensure financial sustainability for medicines, the CEECs implemented, to a greater or lesser extent, several of the policy options available: They applied price regulation for medicines, at least for those (partially) funded by the state at ex-factory price (manufacturer price) as well as at wholesale and retail price level (regulating the maximum allowed profits for distributors and dispensaries) [8–10]. In addition, lower priced generics were more widely used in the CEECs compared to other EU Member States: this might be attributable to a traditionally strong role of local generic industry, but also to demand-side measures such as encouraging doctors to prescribe by International Non-Proprietary Name (INN) and allowing pharmacists to substitute prescribed brands by generics (generic substitution) [11, 12].

Still, investment in health care, including medicines, is known to be lower in the Central and Eastern European region, and several CEECs have been struggling with limited health budgets, making cost-containment a major policy concern [6, 7, 13]. Consequently, out-of-pocket-payments, including informal payments [14–17], considerably increased. Respective austerity measures shifted the financial burden from the state to private households, with a potential risk that vulnerable population groups refrain from using health care services, including getting prescriptions filled and purchasing non-prescribed Over-the-Counter (OTC) medicines [18].

At the same time, limited evidence is available regarding inequalities in medicine use. To our best knowledge, the socioeconomic determinants of medicine use in the CEECs have not yet been explored in detail. Exceptions include Geckova et al. [19], studying socioeconomic differences in health, including the use of prescribed and non-prescribed medicines, of adolescents in Slovakia in 1998, or Gorecka et al. [20] analysing as to whether a socioeconomic gradient existed in the use of (prescribed) cardiovascular medicines at district level in the Czech Republic for the years 1997-2000. Though not studying socioeconomic (in)equalities, the study by Baji et al. [21] is also relevant in this context: Concerns for socioeconomic inequalities were raised following the increase in out-of-pocket payments, including co-payments after the 2007 health care reform in Hungary.

Against this background, our study aims to analyse whether socioeconomic determinants influence medicine use, i.e. both use of prescribed medicines and non-prescribed medicines, in eight CEECs (Bulgaria, Czech Republic, Hungary, Latvia, Poland, Romania, Slovenia, Slovakia). Based on these findings, we discuss to what extent observed inequalities can potentially be explained by current pharmaceutical policies (or a lack of respective policies).

This article is thus first to study inequalities in medicine use in CEECs. To this end, we first present a brief review of the literature on inequalities in health care and, more specifically, medicine use, and, introduce our analytical framework. We perform a quantitative analysis of (in)equalities in medicine use based on household survey data from the first wave of the European Health Interview Survey (EHIS). The findings are interpreted in a policy analysis that follows the developed framework and uses information and research on national pharmaceutical systems.

## Addressing inequalities in medicine use

Following the international literature, equitable access to and equitable use of health care is generally defined as access to and use of health care that is determined by health care need rather than any other criteria such as socioeconomic and demographic factors (e.g. income, education, sex or age) or the macro level institutional factors (e.g. health insurance coverage or co-payments). For medicines, this would require that health care need and no other potential factor determines access to and use of medicines. The empirical and policy oriented literature dealing with (in)equalities in health care revolves around three major issues: measuring the extent of the inequalities, identifying the factors that determine inequalities and addressing measures that help reduce existing inequalities.

Over the past two decades, there has been a vastly growing literature attempting to measure the existence and the extent of socioeconomic inequalities. This first stream of the literature is widely covering developing as well as developed countries (e.g. [22–24]) and has focused on access to general practitioners (GPs) or specialists [25], on access to specific treatments [26], but less on access to hospital care. Existing studies on inequalities in medicine consumption focus on single countries [27] or even regions within countries [28], on specific medicines [29] or specific groups of the population [30], while there is very little cross-country evidence on socioeconomic inequalities in medicines consumption.

The second stream of the literature goes beyond measuring (in)equalities and applies different frameworks to disentangle the factors that determine (in)equalities in access to and use of health care. Much of this work builds on Andersen’s behavioural model [31], i.e. his seminal work on the predictive factors for the use of medical care. While different extensions and adaptations of the original model exist, the approach primarily differentiates between predisposing factors (including demographic factors such as age and gender, socioeconomic factors but also health beliefs or cultural norms), enabling factors (including the financial situation of the user, the means of transportation but also health care system conditions) and the need factor (differentiating between perceived need and evaluated need). Health policies (and pharmaceutical policies) largely fall into the category of contextual enabling factors. In a 2012 review of the application of Andersen’s model in the 1998-2011 period [32], 328 articles have been identified, with more than half referring to the 1995 model [33]. None of the articles identified in this systematic review has dealt with the use of medicines. There are, however, studies on medicine use explicitly or implicitly referring to the ideas of the Andersen model (e.g., [34]).

Another approach to disentangling the factors that determine unequal access to health care services starts from a taxonomic definition of the 5As: availability, accessibility, accommodation, affordability and acceptability [35]. For medicines, this approach has been adapted to include availability, accessibility, acceptability and affordability, with emphasizing quality as a factor relevant in all the four dimensions [36]. In a more recent adaptation for access to health technologies including medicines, for poor populations, availability and affordability have been complemented with architecture and adoption [37]. Bigdeli et al. [38], in an attempt to grasp the complexity of barriers to accessing medicines and to more explicitly address the actors involved, differentiate between the demand side level of individuals, households and communities, health service delivery as the supply side perspective, health sector governance and the national and international context beyond the health sector.

This latter approach puts more emphasis on the health policy perspective. In addition to identifying socioeconomic inequalities in access to and use of health care and in addition to the aforementioned approaches to disentangle the factors that determine existing inequalities, the third stream of the literature on inequalities in health care use deals with the impact of policies on access to and use of medicines or on improving access to health care in general or medicines more specifically. Examples for this body of work are Gemmill et al. [39] analysing the impact of prescription medicines charges on efficiency and equity or Leopold et al. studying the impact of the economic recession on pharmaceutical consumption in eight European countries [40] and the impact of the policy policies on the use of antipsychotic medicines for Finland and Portugal [41]. In general, the more policy oriented literature is broad and often aimed at very specific health policy concerns. But often it lacks a coherent framework for analysis. For our study, we follow a WHO approach for collective action towards equitable access to essential medicines [42], differentiating between four key actions: rational selection and use of essential medicines, affordable prices, sustainable financing, and reliable supply systems.

With a view to the aim of this study, discussing the impact of pharmaceutical policies on (in)equalities in the use of medicines, and in combining the above cited literature, we use four key dimensions for the analysis: (1) sustainable funding, (2) affordability, (3) rational selection and use of medicines and (4) availability and accessibility. As depicted in Table 1, the first two dimensions are dealing with financing, (1) on the macro level, (2) on the micro level, the other two dimension are related to issues of delivery, (3) on the macro level, (4) on the micro level.

**Table 1:** Pharmaceutical policies and equitable access to medicines: a framework for analysis

	Macro level	Micro level
FINANCING	Sustainable funding	Affordability
	*Addressing financial issues on macro levels: the availability of funding for health care in general and the level of pharmaceutical spending more specifically*	*Addressing financial issues on micro levels: who pays and how much, thus the role of public and private payments for pharmaceuticals*
	Categories of indicators: type of health care system, total health expenditure, pharmaceutical expenditure	Categories of indicators: private health expenditure, private pharmaceutical expenditure, co-payments, informal payments, medicine prices, VAT on medicines, policies addressing the poor
DELIVERY	Rational selection and use	Availability and accessibility
	*Addressing delivery on macro levels: which medicines, at least potentially, are made available, rational prescribing, dispensing and use of medicines in the health care system*	*Addressing delivery on micro levels: the issue of supply meeting demand in terms of volume and reachability given distances and travel resources*
	Categories of indicators: INN prescribing, generic market, reference price system, prescription monitoring, treatment guidelines	Categories of indicators: number of medicines, pharmacy density, OTC sale

Note: A definition of all the indicators chosen is given in Table 5 which provides a comparative overview for the eight study countries.

## Methods

### Quantitative analysis

We draw on cross-sectional data from the first wave of the European Health Interview Survey (EHIS), collected between 2006 and 2009 in 19 EU Member States [43]. This study focuses on CEECs. Countries in this region, due to their weaker economic situation compared to Western European countries, risk substantial socioeconomic inequalities. Eight (Bulgaria, Czech Republic, Hungary, Latvia, Poland, Romania, Slovenia, Slovakia) of the ten CEECs that were EU Member States at the time of the EHIS survey were included in our study (see Table 2 for an overview of the dataset); Estonia and Lithuania were excluded due to limited data and non-inclusion in the EHIS survey, respectively. Generally, the data focussed on the non-institutionalized population, but in the Czech Republic also the institutionalized population (living in nursing homes and convents/monasteries) was included in the survey [44]. Since this, however, only concerns less than an estimated 0.5 % of this country’s sample population (Š Daňková 2014, pers. comm., 6 October), we hardly expect this to affect our results. Finally, the EHIS dataset covers the population above age 15, however, we restrict our analysis to adults (aged 18 and over) as it is assumed that the development of an individual socioeconomic status may be expected from around this age onwards.

**Table 2:** Data sources

	BG	CZ	HU	LV	PL	RO	SI	SK
Fieldwork	10/2008–11/2008	06/2008–10/2008	09/2009–10/2009	09/2008–12/2008	10/2009–12/2009	05/2008–06/2008	10/2007–11/2007	09/2009–10/2009
Response rate in %	74	56	81	72	72	89	68	66
Sample size (age 15+)	5,661	1,995	5,051	6,458	35,100	18,172	2,188	4,972

Note: *BG* Bulgaria, *CZ* Czech Republic, *HU* Hungary, *LV* Latvia, *PL* Poland, *RO* Romania, *SI* Slovenia, *SK* Slovakia

Source: Eurostat [44]

To determine the association between socioeconomic status and pharmaceutical consumption, we carried out multinomial multivariate logistic regression analyses (Stata 13.1). In these models, the probability was estimated of falling into certain categories compared to the reference group. To check the statistical significance of the calculated relative-risk ratios (RRR), z-statistics were used. Only results with *p* < 0.05 were considered statistically significant and thus discussed further. McFadden’s R^2^ is reported as measure of model fit.

A detailed description of the variables included in the regression analysis can be found in Table 3. The dependent variable is based on the information on the consumption of non-prescribed medicines (only), consumption of prescribed medicine (only) and consumption of both types of medicines versus no medicine use. For an unbiased gender comparison, use of female-specific medicines (contraceptive pills; hormones for menopause; based on an additional survey question) was excluded from the analysis. As independent variables of interest, three socioeconomic indicators were included [45]. First, employment status was grouped into employed (reference group), retired, unemployed or disabled and in training or at home. Second, the highest level of education completed was measured based on the International Standard Classification of Education (ISCED) 1997 and distinguishes between ISCED ≤ 2, ISCED 3-4 and ISCED 5-6. Third, the income level was based on the range of the monthly net household income against the values of the national deciles’ limits and categorized into quintiles (reference group: first quintile). To control for demographic characteristics, we included age and sex, with the youngest and males as reference groups. Finally, since a socioeconomic gradient in health disadvantaging the poor is also well-established in CEE regions [46], we introduced dummy variables for chronic conditions (no chronic conditions as reference group) and self-assessed health (good as reference group).

**Table 3:** Variable definitions

Variable	Survey question	Subcategories (as used in the analysis) and reference group
Medicine use	During the past two weeks, have you used any medicines (including dietary supplements such as herbal medicines or vitamins) that were prescribed or recommended for you by a doctor?	• None; reference group • Non-prescribed • Prescribed • Both
	During the past two weeks, have you used any medicines or dietary supplement or herbal medicines or vitamins not prescribed or recommended by a doctor?	
Employment status	How would you define your current labour status?	• Employed (working for pay or profit); reference group • Retired (in retirement or early retirement or has given up business) • Unemployed or disabled (unemployed; permanently disabled)^a^ • In training or at home (pupil, student, further training, unpaid work experience)
Highest education	What is the highest education leaving certificate, diploma or education degree you have obtained? Please include any vocation training.	• ISCED ≤ 2 (no formal education or below ISCED 1; primary education; lower secondary education); reference group • ISCED 3-4 (upper secondary education; post-secondary but non-tertiary education) • ISCED 5-6 (first stage of tertiary education; second stage of tertiary education)
Income quintile	Perhaps you can provide the appropriate range [instead of the household’s total net income per month]. Would you (please look at this card and) tell me which group represents your household’s total net monthly income from all these sources after deductions for income tax, National Insurance etc. Is it …	• First quintile; reference group • Second quintile • Third quintile • Fourth and fifth quintile
Age	Age of the person at the moment of the interview	• 18–29 years • 30–39 • 40–49 • 50–59 • 60–69 • 70+
Gender	Sex	• Male; reference group • Female
Chronic conditions	Do you have any longstanding illness or [longstanding] health problem? [By longstanding, I mean illnesses or health problems which have lasted, or are expected to last, for 6 months or more.]	• No; reference group • Yes
Self-assessed health	How is your health in general? Is it …	• Good (very good, good); reference group • Poor (very bad, bad, fair)

Note: ^a^As pointed out by an anonymous reviewers, it can be argued that unemployed and permanently disabled people differ in terms of health and medicine use and should thus be analysed separately. However, as both groups individually make for a small proportion of the sample and thus only very few observations would be left for the subgroup analyses (in the most extreme case 0 observations and in the majority of cases, less than 20 observations, which, for Eurostat confidentially reasons would need to be left blank), it was decided to analyse them as one subgroup.

Source: Data provided by Eurostat [80]; presentation by the authors

An overview of the descriptive information for the individual countries is given in Table 4.

**Table 4:** Descriptive information

	BG		CZ		HU		LV		PL		RO		SI		SK	
	*n*	%	*n*	%	*n*	%	*n*	%	*n*	%	*n*	%	*n*	%	*n*	%
Medicine use	5,475	100.0	1,866	100.0	4,899	100.0	6,114	100.0	33,416	100.0	17,464	100.0	2,028	100.0	4,737	100.0
None	2,185	39.9	508	27.2	1,558	31.8	2,161	35.3	7,714	23.1	9,742	55.8	705	34.8	1570	33.1
Non-prescribed	792	14.5	357	19.1	750	15.3	1,308	21.4	7,036	21.1	1,284	7.4	409	20.2	941	19.9
Prescribed	1,393	25.4	529	28.3	1,557	31.8	1,381	22.6	7,022	21.0	5,013	28.7	595	29.3	1,051	22.2
Both	666	12.2	454	24.3	1,031	21.0	1,084	17.7	7,835	23.4	1,351	7.7	318	15.7	1,157	24.4
Missing	439	8.0	18	1.0	3	0.1	180	2.9	3,809	11.4	74	0.4	1	0.0	18	0.4
Employment status	5,475	100.0	1,866	100.0	4,899	100.0	6,114	100.0	33,416	100.0	17,464	86.5	2,028	85.9	4,737	89.9
Employed	2,682	49.0	995	53.3	2,462	50.3	3,324	54.4	15,836	47.4	8,261	47.3	1,005	49.6	2,798	59.1
Retired	1,875	34.2	594	31.8	1,322	27.0	1,551	25.4	9,263	27.7	6,132	35.1	553	27.3	991	20.9
Unemployed, disabled	631	11.5	104	5.6	740	15.1	602	9.9	4,002	12.0	722	4.1	184	9.1	471	9.9
In training, at home	287	5.2	173	9.3	375	7.7	637	10.4	4,315	12.9	2,349	13.0	286	14.1	477	10.1
Missing	0	0.0	0	0.0	0	0.0	0	0.0	0	0.0	0	0.0	0	0.0	0	0.0
Highest education	5,475	100.0	1,866	100.0	4,899	100.0	6,114	100.0	33,416	100.0	17,464	100.0	2,028	100.0	4,737	100.0
ISCED ≤ 2	1,830	33.4	292	15.6	1,289	26.3	1,760	28.8	7,741	23.2	6,381	36.5	903	44.5	525	11.1
ISCED 3-4	2,754	50.3	1,370	73.4	2,728	55.7	3,155	51.6	20,257	60.6	9,568	54.8	955	47.1	3,311	69.9
ISCED 5-6	889	16.2	196	10.5	877	17.9	1,196	19.6	5,418	16.2	1,515	8.7	170	8.4	901	19.0
Missing	2	0.0	8	0.4	5	0.1	3	0.0	0	0.0	0	0.0	0	0.0	0	0.0
Income quintile	5,475	100.0	1,866	100.0	4,899	100.0	6,114	100.0	33,416	100.0	17,464	100.0	2,028	100.0	4,737	100.0
1	1,384	25.3	189	10.1	1,048	21.4	201	3.3	3,530	10.6	9,592	54.9	260	12.8	559	11.8
2	577	10.5	217	11.6	890	18.2	2,101	34.4	5,860	17.5	6,190	35.4	358	17.7	947	20.0
3	787	14.4	383	20.5	1,015	20.7	2,809	45.9	5,915	17.7	595	3.4	281	13.9	1,034	21.8
4–5	2,452	44.8	683	36.6	1,537	31.4	1,003	16.4	10,884	32.6	171	1.0	611	30.1	1,603	33.8
Missing	275	5.0	394	21.1	409	8.3	0	0.0	7,227	21.6	916	5.2	518	25.5	594	12.5
Age	5,475	100.0	1,866	100.0	4,899	100.0	6,114	100.0	33,416	100.0	17,464	100.0	2,028	100.0	4,737	100.0
18–29	767	14.0	344	18.4	841	17.2	1,328	21.7	6,673	20.0	2,717	15.6	420	20.7	1,121	23.7
30–39	751	13.7	344	18.4	919	18.8	963	15.8	5,261	15.7	2,985	17.1	338	16.7	965	20.4
40–49	894	16.3	243	13.0	756	15.4	984	16.1	5,361	16.0	2,840	16.3	355	17.5	800	16.9
50–59	1,061	19.4	320	17.1	933	19.0	972	15.9	6,804	20.4	3,320	19.0	380	18.7	837	17.7
60–69	948	17.3	326	17.5	732	14.9	882	14.4	4,548	13.6	2,593	14.8	279	13.8	577	12.2
70+	1,054	19.3	289	15.5	718	14.7	985	16.1	4,769	14.3	3,009	17.2	256	12.6	437	9.2
Missing	0	0.0	0	0.0	0	0.0	0	0.0	0	0.0	0	0.0	0	0.0	0	0.0
Gender	5,475	100.0	1,866	100.0	4,899	100.0	6,114	100.1	33,416	100.0	17,464	100.0	2,028	100.0	4,737	100.0
Male	2,588	47.3	891	47.7	2,225	45.4	2,692	44.1	15,295	45.8	8,202	47.0	948	46.8	2,271	47.9
Female	2,887	52.7	975	52.3	2,674	54.6	3,422	56.0	18,121	54.2	9,262	53.0	1,080	53.3	2,466	52.1
Missing	0	0.0	0	0.0	0	0.0	0	0.0	0	0.0	0	0.0	0	0.0	0	0.0
Chronic conditions	5,475	100.0	1,866	100.0	4,899	100.0	6,114	100.0	33,416	100.0	17,464	100.0	2,028	100.0	4,737	100.0
No	3,020	55.2	951	51.0	1,430	29.2	3,413	55.8	15,705	47.0	11,143	63.8	1,245	61.4	1,957	41.3
Yes	2,429	44.4	904	48.4	3,467	70.8	2,692	44.0	17,557	52.5	6,206	35.5	779	38.4	2,767	58.4
Missing	26	0.5	11	0.6	2	0.0	9	0.1	154	0.5	115	0.7	4	0.2	13	0.3
Self-assessed health	5,475	100.0	1,866	100.0	4,899	100.0	6,114	100.0	33,416	100.0	17,464	100.0	2,028	100.0	4,737	100.0
Good	3,099	56.6	1,161	62.2	2,529	51.6	2,661	43.5	16,287	48.7	11,252	64.4	1,237	61.0	2,975	62.8
Bad	1,950	35.6	703	37.7	2,369	48.4	3,275	53.6	13,346	39.9	6,204	35.5	789	38.9	1,756	37.1
Missing	426	7.8	2	0.1	1	0.0	178	2.9	3,783	11.3	8	0.0	2	0.1	6	0.1

Source: Data provided by Eurostat [43]; calculation and presentation by the authors

### Pharmaceutical policy analysis

The analysis of pharmaceutical policies as potential co-determinant of inequalities in medicine use builds on an analytical framework based on the WHO approach for collective action [42] (see Addressing inequalities in medicine use and Table 1). We identified four key dimensions for the analysis, (1) sustainable funding, (2) affordability, (3) rational selection and use of medicines and (4) availability and accessibility. For each of the four dimensions, we developed a set of indicators (see Tables 1 and 5).

**Table 5:** Pharmaceutical policy framework – Indicators for equitable access to essential medicines

Indicator	BG	CZ	HU	LV	PL	RO	SI	SK
Reference year	2008	2008	2009	2008	2009	2008	2007	2009
Sustainable funding								
HC system	SHI	SHI	SHI	SHI	SHI	SHI	SHI	SHI
TPE in % of THE	33.4 % (0.0 pp)	20.4 % (-4.4 pp)	32.5 % (+1.5 pp)	19.4 % (-2.6 pp)	22.9 % (-4.3 pp)	25.0 % (-3.2 pp)	19.6 % (-1.1 pp)	26.6 % (-3.2 pp)
TPE share (CEE-10 comparison)	+9.0 pp	−4.0 pp	+6.7 pp	−5.0 pp	−1.5 pp	+0.6 pp	- 6.0 pp	+3.2 pp
TPE per capita	€ 109.9 (+50.3 %)	€ 205.6 (+17.1 %)	€ 229.4 (+0.8 %)	€ 134.3 (+66.6 %)	€ 134.5 (+11.7 %)	€ 92.2 (+59.1 %)	€ 264.0 (+12.0 %)	€ 283.5 (+56.6 %)
TPE/capita (CEE-10 comparison)	−39.1 %	+14.0 %	+35.3 %	−25.6 %	−16.5 %	−48.9 %	+56.9 %	+47.0 %
Affordability								
Private HE/THE	41.8 % (+6.5 %)	17.5 % (+37.5 %)	34.3 % (+13.5 %)	37.4 % (-12.7 %)	28.2 % (-6.0 %)	18.0 % (-6.2 %)	28.1 % (+4.6 %)	34.3 % (+8.3 %)
Private HE/THE (CEE-10 compar.)	+13.6 pp	−10.8 pp	+4.7 pp	+9.2 pp	- 0.1 pp	−10.3 pp	−2.2 pp	+4.0 pp
Private PE/TPE	81.7 % (+4.8 %)	38.4 % (+56.9 %)	51.6 % (37.7 %)	62.2 % (-9.4 %)	61.4 % (0 %)	55.0 % (+4.8 %)	40.1 % (+2.9 %)	30.2 % (+11.3 %)
Private PE/TPE (CEE-10 comp.)	+27.8 pp	−15.5 pp	−2.9 pp	+8.3 pp	+7.8 pp	+1.2 pp	−13.7 pp	−25.2 pp
Informal pays	Yes, extensive	Yes	Yes	Yes, extensive	Yes	Yes, large scale	No	Yes
Medicine prices	n.a.	€ 5.09 (-71.4 %)	€ 6.91 (-61.2 %)	€ 4.23 (-76.2 %)	€ 4.54 (-74.5 %)	n.a.	€ 12.34 (-30.8 %)	n.a.
Co-pays (out-patient)	No PF	PF	PF	No PF	PF for some indications	No PF	No PF	PF
	%: 0 %, >25 %, >50 %	No fixed R rates	%: 0 %, 10 %, 30 %; 25 %, 45 %, 75 %	%: 0 %, 10 %, 25 %, 50 %, 25 %	%: 0 %, 30 %, 50 %	%: 0 %, 10 %, 50 %	%: 0 %, 25 %, 75 %	%: no fixed R rates
	No D	No D	No D	No D	No D	No D	No D	No D
Changes in co-pays	No	Yes	Yes, increase	No, but planned (90 % R rate to be abolished)	No since mid-1990-ties	No	No	Yes, decrease in PF in 10/2006
Co-pay exempt.	None	None	Yes	Yes	Yes	Yes, see comment	Yes	Yes
De-listings	No, see comment	Not known	Yes, see comment	See comment	Yes	See comment	No	No major delisting
Co-pays (in-patient)	No co-payment	No co-payment	No co-payment	No co-payment	No co-payment	No co-payment	No co-payment	No co-payment
VAT on medicines	20 % (20 %)	9 % (19 %)	5 % (20 %)	5 % (18 %), planned increase from 1/2009	7 % (22)	9 % (19 %)	8.5 % (20 %)	10 % (19 %)
OTC market	22 %	26.8 %	15.8 %	n.a.	25.8 %	22.4 %	6.7 %	25.8 %
Availability and accessibility								
No. of medicines	5,830^2006^	7,880^2006^	5,525 ^2006^	n.a.	(8,089^2005^)	6,711^2007^	2,791^2006^	19,693 ^November 2006^
	4,481^2006^	4,130^2006^	3,144^2006^	5,714^2007^	4,275^2006^	≈4,000^2007^	n.a.	19,320^November 2006^
	1,598	6,988^2006^	2,886^2006^	2,337^2007^	2,749^2006^	5,791^2007^	2,660^2006^	17,804^November 2006^
	See comment		See comment	See comment	See comment	See comment	See comment	See comment
Pharmacies	4,299/1,795^2006^	2,520/4,096^2007^	≈2,500/≈4,000^2010^	899/2,209^2007^	10,632/3,585^2007^	5,660/3,733^2007^	271/7,384^2005^	1,523/3,528^Oct. 2006^
OTC sale outside pharmacies permitted	Yes	Yes	Yes	No	No	Yes, but pharmacist must be present	Yes	No
Rational selection and use of medicines								
INN prescribing	Yes, indicative	Yes, indicative	Yes, indicative	Yes, indicative	Yes, indicative	Yes, indicative	Yes, indicative	Yes, indicative
Generic substitution	No, not allowed	Yes, indicative	Yes, indicative	Yes, obligatory	Yes, indicative	Yes, indicative	Yes, indicative	Yes, obligatory
Ref. price syst.	Yes, ATC 5	Yes, ATC 4 + 5	Yes, ATC 4 + 5	Yes, ATC 3,4 + 5	Yes, ATC 3,4 + 5	Yes, ATC 5	Yes, ATC 4	Yes, ATC 4 + 5
Generic market	See comment	55 %/35 %^2005^	42 %/26 %^2005^	No data	No data	≈70 %/≈40 %^e^	70 %/38 %^2005^	65 %/48 %^2005^
Prescription monitoring	Yes, but limited	Yes	Yes, but limitations in enforcement	Yes	Yes, but limitations in enforcement	Yes, but limitations in enforcement	Yes	Yes

Indicators:

Reference year: data in this table refer to the year of the household survey unless indicated differently

HC system: indicates the type of health care system: Semaskho system, social health insurance (SHI) or National Health Service (NHS)

TPE in % of THE: Total pharmaceutical expenditure (TPE) in % of total health expenditure (THE) in the year of the survey; in brackets: change in the share of TPE in % of THE in percentage points compared to 3 years earlier

TPE share (CEE-10 comparison): Difference of the share of TPE in % of THE in the surveyed country in 2008 compared to CEE-10 average (Bulgaria, Czech Republic, Estonia, Latvia, Lithuania, Hungary, Poland, Romania, Slovenia, Slovakia) in 2008, expressed in percentage points

TPE per capita: Total pharmaceutical expenditure (TPE) per capita in the year of the survey, in Euro; in brackets: change in TPE per capita compared to 3 years earlier, expressed in per cent)

TPE/capita (CEE-10 comparison): Difference of the TPE per capita in the surveyed country in 2008 compared to CEE-10 average in 2008, expressed in per cent

Private HE/THE: Private health expenditure (HE) in % of total health expenditure (THE) in the year of the survey (unless BG – 2007 data); in brackets: change in the share of private HE in % of THE in per cent compared to 3 years earlier

Private HE/THE (CEE-10 compar.): Difference of the share of private HE in % of THE in the surveyed country in 2008 compared to CEE-10 average in 2008 (unless BG – 2007 data), expressed in percentage points

Private PE/TPE: Private pharmaceutical expenditure (PE) in % of total pharmaceutical expenditure (TPE) in the year of the survey; in brackets: change in the share of private PE in % of TPE in per cent compared to 3 years earlier

Private PE/TPE (CEE-10 comp.): Difference of the share of private PE in % of TPE in the surveyed country in 2008 compared to CEE-10 average in 2008, expressed in percentage points

Informal pays: Indications of informal payments in health care

Medicine prices: Average pharmacy retail price in € per pack in the total out-patient market in 2006; differences in % to average of EU-15

Co-pays (out-patient): Co-payments for medicines in the out-patient sector: PF = prescription fee, % = percentage co-payment rates (for reimbursable medicines different co-payment rates in per cent of the price of product apply, usually linked to the severity of the disease and therapeutic benefit of the medicine), D = deductible (upfront initial out-of-pocket payment up to a fixed amount for a service or over a defined period of time; then the rest of the cost is covered by a public party payer). Percentage co-payments due to the reference price system (see below indicator ‘Ref. pricing syst.’) are not considered.

Changes in co-pays: Changes in co-payments in the years before the survey

Co-pay exempt.: Mechanisms for vulnerable groups (e.g. exemptions, reductions) from co-payments in the out-patient sector

De-listings: Exclusion of medicines from reimbursement in the years before the survey

Co-pays (in-patient): Co-payments for medicines in the in-patient sector

VAT on medicines: Value-added tax (VAT) rate on medicines; in bracket: standard VAT rate

OTC market: Relevance of the Over-the-Counter (OTC) market expressed by the share of sales in total non-prescription market as per cent of total pharmaceutical market sales in the years of the EHIS survey (at consumer price level, unless indicated differently)

No. of medicines: Number of medicines authorized, medicines on the market, and prescription-only medicines. Counted incl. different pharmaceutical forms and dosages, excl. different pack sizes unless indicated differently

Pharmacies: Number of community pharmacies, and inhabitants per pharmacy served

OTC sale outside pharmacies permitted: Information as to whether the sale of Over-the-Counter (OTC) medicines was permitted outside pharmacies, or not

INN prescribing: Information as to whether prescribing by International Non-Proprietary Name (INN) was permitted, and if yes, whether the policy was voluntary (indicative) or mandatory for the prescriber

Generic substitution: Information as to whether the practice of substituting a medicine, whether marketed under a trade name or generic name (branded or unbranded generic), with a less expensive medicine (e.g. branded or unbranded generic), often containing the same active ingredient, was permitted, and if yes, whether the policy was voluntary (indicative) or mandatory for the pharmacist

Ref. price syst.: Information as to whether a reference price system was in place, i.e. a reimbursement policy, in which identical or similar medicines are clustered – the public payer reimburses a defined maximum amount (reference price) for all medicines of the cluster, and patients have to co-pay the remainder up to the pharmacy retail price gross), and its clustering: ATC-5: clustering of same active ingredients or groups of active ingredients, ATC-4: clustering of medicines of the same therapeutic group, clustering of medicines of the same pharmacological subgroup

Generic market: Generic market share (in value and in volume) in the out-patient sector

Prescription monitoring: Information as to whether prescription monitoring was performed

*Abbreviations*: *ATC* Anatomical Therapeutic Chemical classification system, *D* deductible, *HE* health expenditure, *OTC* Over-the-Counter medicines, *pp* percentage points, *PE* pharmaceutical expenditure, *PF* prescription fee, *R* reimbursement, *THE* total health expenditure, *TPE* total pharmaceutical expenditure, *VAT* value-added tax

Notes:

BG:

Private HE / THE and Private HE / THE (CEE-10 compar.): no data for private health expenditure in 2008 available; 2007 data used instead: change indicated for the years 2004-2007

Changes in co-pays: Increase of percentage co-payments for some medicines, treating Parkinson, osteoporosis, Glaucoma, etc. took place after the EHIS survey, in 2010

De-listings: No de-listings reported but constant increase of medicines included in the positive list during 2007

No. of medicines: Different data sources and counting methods for medicines authorized and on the market compared to prescription-only medicines. The latter were counted per brand name, excl. different pharmaceutical forms, dosages and pack sizes. Counted according to that method, the number of the medicines on the market were 4,299, thus resulting in a share of prescription-only medicines of the medicines on the market of around 37 %.

Pharmacies: High regional disparities in the density of pharmacies, concentration in larger cities

Generic market: No data on generic market shares available. 74 % of the medicines on the market are generics.

CZ:

Co-pays (out-patient): Percentage co-payment rates result from application of the reference price system

Changes in co-pays: In 2008 (year of the survey), a prescription fee was introduced, as well as further co-payments in health care (not medicines related) such as a co-payment for visit to a doctor

Pharmacies: High regional disparities in the density of pharmacies, concentration in larger cities

HU:

Co-pays (out-patient): Percentage co-payment rates of 0, 10 and 30 % for out-patient medicines for specific indications, and of 25, 45 and 75 % for all other reimbursable medicines in the out-patient sector

Co-pay exempt.: Exemptions from co-payment for socially disadvantaged persons. 100 % reimbursement for patients with long-term illness, however, the prescription fee had also to be paid by them

De-listings: A few de-listings, and changes in reimbursement categories (pharmaceutical groups were granted a lower percentage reimbursement rate) at large scale since 2006/2007

No. of medicines: Counted per brand name, excl. different pharmaceutical forms, dosages and pack sizes

Pharmacies: In addition to pharmacies, around 370 doctors are also allowed to dispense prescription-only medicines

Prescription monitoring: Limitations particularly related to off-patent medicines: guidelines for cost-effective prescribing are frequently not followed, no audit or feed-back of the social health insurance on generic prescribing by doctors

LV:

De-listings: De-listings were not explicitly reported but took place most probably: since 2005 major changes in the reimbursement list were reported, the principle of a limited number of medicines in the positive list was in place, and regular reimbursement reviews were performed. Medicines were included in the positive list for a period of 2 years, then the marketing authorization holder had to apply for re-inclusion.

No. of medicines: Number of medicines authorized not included since medicines are only counted per trade name

Pharmacies: Including 100 branch pharmacies, availability of pharmacies varied between towns and rural areas

PL:

OTC market: At manufacturer price level; refers to self-medication market

No. of medicines: Data on number of prescription-only medicines includes hospital-only medicines. In 2009, 3,380 medicines (counted including different pharmaceutical forms, dosages and pack sizes) were on the reimbursement list. Data on authorized medicines have to be interpreted with caution due to a different counting method: counted including different dosages, pharmaceutical forms and pack sizes.

Pharmacies: High regional disparities in the density of pharmacies, concentration in larger cities

RO:

Co-pay exempt.: Exemptions from co-payments are made for children, students and pregnant women as well as war veterans and disabled people on low income

De-listings: Quarterly updates of the reimbursement lists; no confirmed information of major de-listings (however number of medicines on the reimbursement list is, in general, rather low)

No. of medicines: 1,247 of the 5,791 prescription-only medicines were hospital-only medicines

Pharmacies: High regional disparities in the density of pharmacies, concentration in larger cities

Generic market: Estimate for the year 2007

SI:

Co-pays (out-patient): Co-payments covered by voluntary insurance that around 90 % of the population concluded

Co-pay exempt.: Exemptions from co-payments for medicines for prevention, for defined social groups (such as people under 18 years) or for the treatment of specific diseases (e.g. HIV/AIDS, diabetes)

De-listings: No major wave of de-listings reported; on the contrary, some new medicines could be included in the positive lists following changes in medicine prices due to change in pricing methodology

OTC market: At wholesale price level, refers to self-medication market

No. of medicines: Counted including different dosages, pharmaceutical forms and pack sizes

Pharmacies: Some OTC medicines were ‘pharmacy-only’, whereas others were permitted to be sold in ‘specialised stores’ outside pharmacies

SK:

No. of medicines: Counted including different pharmaceutical forms, dosages and pack sizes; homeopathic medicines excluded

Sources: Indicator 2: [7, 10, 81–89]; Indicators 3-10: [49]; Indicator 11: [14–16, 63, 83, 86, 88–100]; Indicator 12: [68]; Indicators 13-16, 18, 20, 22-24, 26, 27: [9, 63, 101–111]; Indicator 17: [112–117]; Indicator 19: [53–55]; Indicator 21: pharmacy data and information on disparities: [7, 9, 63, 82, 84, 86, 102, 103, 105, 111, 118, 119], population data: [49]; Indicator 25: RO: [12, 63]

To fill these indicators with country specific information, we drew on several data sources: Background information and data for the policy analysis were taken from the literature, predominantly grey literature. Search terms included ‘medicines’, ‘pharmaceuticals’, ‘equity’, ‘access’ and the name of the country. We focussed on the period around the EHIS survey, and three years before, and also literature with overall relevant information, e.g. related to the transition process, was included. Country reports about national pharmaceutical systems were a key source of information, in particular the PPRI (Pharmaceutical Pricing and Reimbursement Information) Pharma Profiles (available for five of the eight CEECs), the Health Systems in Transition reports by the European Observatory on Health Systems and Policies as well as country reports produced by the Organisation for Economic Co-operation and Development (OECD) and by the Austrian Public Health Institute (references listed below Table 5 and below the additional table [see Additional file 1]). Since some of the reports did not refer to the exact years of the survey, unpublished information from the PPRI network was used as supplementary source [47, 48]. Health and pharmaceutical expenditure data were retrieved from the Eurostat database [49].

Based on the information and data collected for the countries, we produced eight brief country posters [see Additional file 1]) and an overview table (Table 5). Following methods of policy analysis research [50], we used the country specific information and data to understand whether the identified socioeconomic (in)equalities in medicine use were impacted by the existing implementation degree of pharmaceutical policies. In the analysis, we interpreted ‘prescribed medicines’ as medicines funded, at least partially, by state (independently from their prescription status: both prescription-only and non-prescription medicines), whereas ‘non-prescribed medicines’ were considered as non-funded self-medication.

## Results

As can be seen from the descriptive information for the individual countries (Table 4), the proportion of people reporting to take no medicine at all ranged between 23.1 % (Poland) and 39.9 % (Bulgaria) in all countries except for Romania, in which 55.8 % reported no medicine consumption. Romania also holds a special position regarding non-prescribed medicine use (7.4 %), which lied between 14.5 % (Bulgaria) and 21.4 % (Latvia) in the remaining countries. Prescribed medicines (only) were taken by around 21.0 % (Poland) and 31.8 % (Hungary) of the surveyed populations.

### Socioeconomic (in)equalities in medicine use

**Fig. 1:**
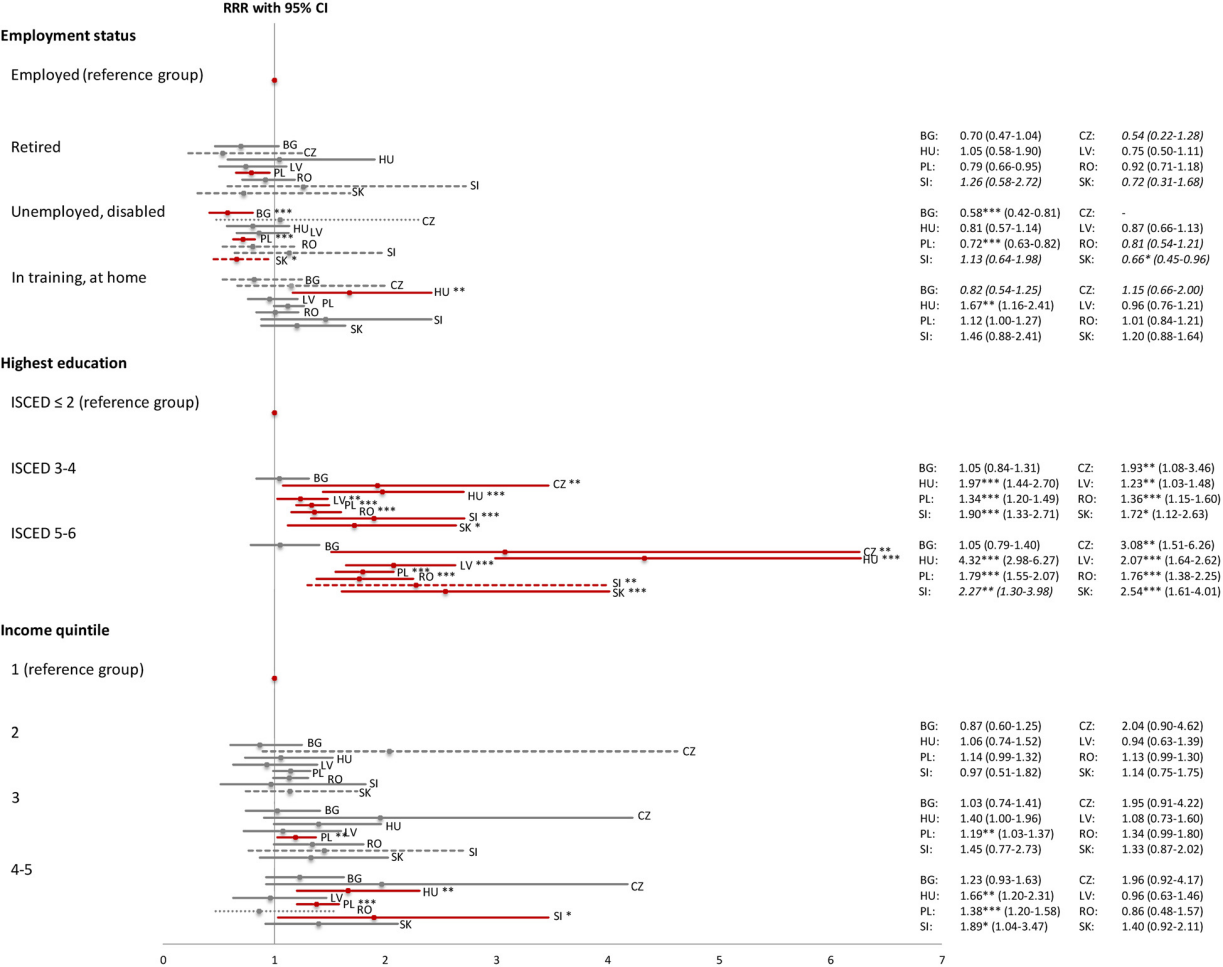
Socioeconomic determinants of non-prescribed medicine use (versus no medicine use). Note: Graphical illustration of the full results [see Additional file 2] regarding non-prescribed medicine use for socioeconomic indicators; RRR = multivariate relative risk ratio, CI = 95 % confidence interval, grey line: not significant, red line: *** = significant at 0.01 %, ** = significant at 0.1 %, * = significant at 5 %. Results based on less than 20 observations: tightly dashed line and exact numbers omitted; results based on 20-49 observations: widely dashed line and exact numbers in italics. Source: Data provided by Eurostat [43]; calculation and presentation by the authors

**Fig. 2:**
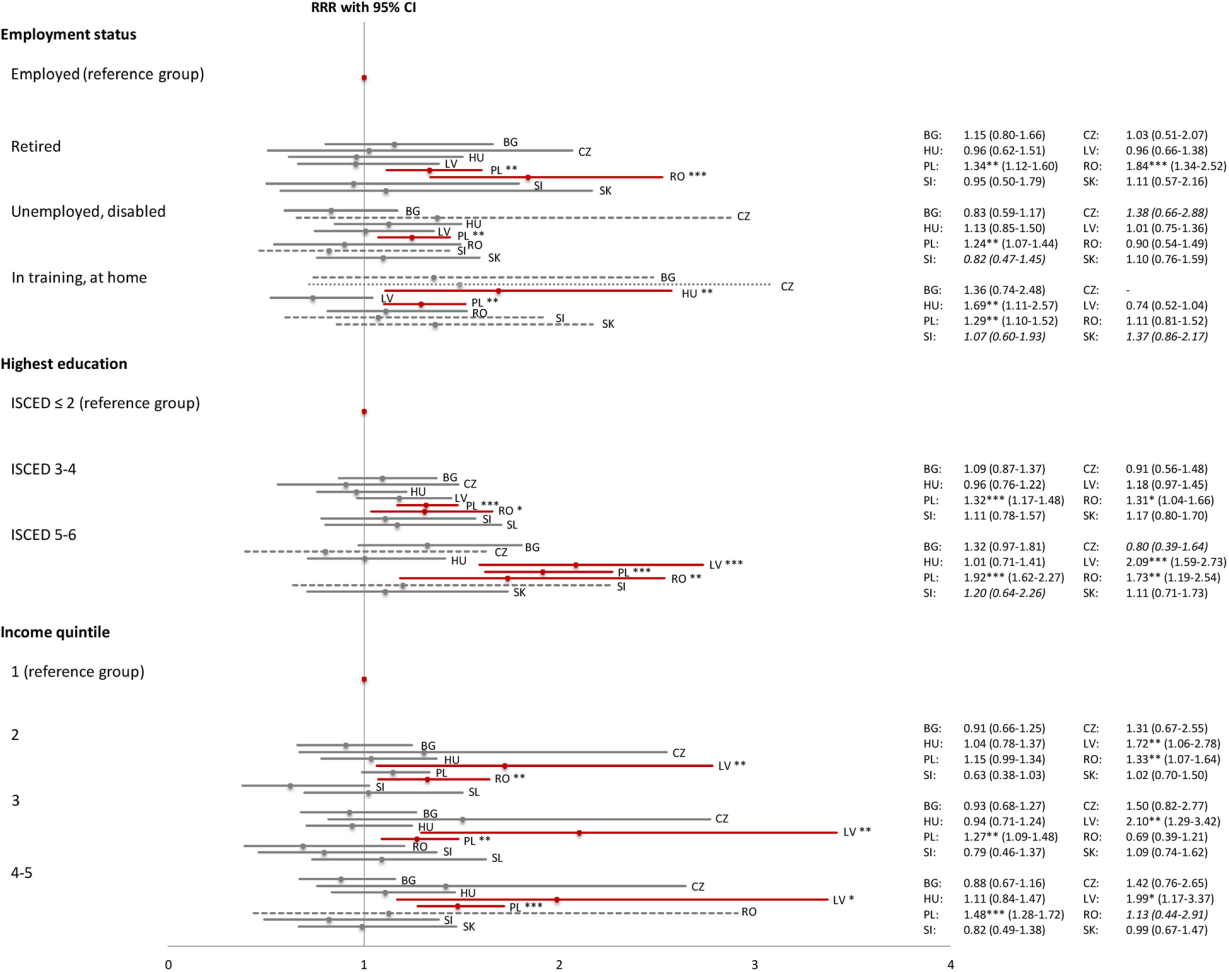
Socioeconomic determinants of prescribed medicine use (versus no medicine use). Note: Graphical illustration of the full results [see Additional file 2] regarding prescribed medicine use for socioeconomic indicators; RRR = multivariate relative risk ratio, CI = 95 % confidence interval, grey line: not significant, red line: *** = significant at 0.01 %, ** = significant at 0.1 %, * = significant at 5 %. Results based on less than 20 observations: tightly dashed line and exact numbers omitted; results based on 20-49 observations: widely dashed line and exact numbers in italics. Source: Data provided by Eurostat [43]; calculation and presentation by the authors

Overall, in the regression analyses we find that whenever there is a socioeconomic gradient in medicine consumption to be observed in a country, it will in virtually every case favour the well-off, as shown in Figs. 1 and 2, respectively. The full results including all variables of the country-specific multinomial logistic regression analyses are presented in an additional table [see Additional file 2].

For non-prescribed medicine use (versus no medicine use), individuals with higher education and/or income had a higher chance of taking such medicines in seven of the eight CEECs: In Hungary, for instance, individuals in the highest education group were estimated to be four times more likely to take non-prescribed medicines (RRR = 4.32, 95 % CI = 2.98–6.27) while the relative risk for people in high income households increases by a factor of 1.66 (95 % CI = 1.20–2.31), respectively, given the other variables in the model are held constant. In Poland, on the other hand, the observed gradient was comparably less pronounced (highest education: RRR = 1.79, 95 % CI = 1.55–2.07; highest income: RRR = 1.38, 95 % CI = 1.20–1.58). Only in Bulgaria, neither education nor income played any role for non-prescribed medicine use, but the unemployed and disabled were attributed a lower consumption likelihood.

For prescribed medicines, in contrast, a statistically significant socioeconomic gradient could be identified in three economies only, i.e. Latvia, Poland, Romania: The increased likelihood of the highest education groups in these countries (Latvia: RRR = 2.09, 95 % CI = 1.59–2.73; Poland: RRR = 1.92, 95 % CI = 1.62–2.27; Romania: RRR = 1.73, 95 % CI = 1.19–2.54) is similar in magnitude, whereas in terms of the highest income groups, Latvia showed the most pronounced effect with an RRR close to 2 (RRR = 1.99, 95 % CI = 1.17–3.37).

Regarding the influence of demographic characteristics on medicine use [see Additional file 2], females were more likely to take (any) medicines in general, whereas advanced age was particularly relevant for prescribed pharmaceutical consumption, which is consistent with the literature [51]. Such prescribed medicine use was also highly determined by health characteristics, especially by longstanding illnesses.

### Pharmaceutical policies

Details on the pharmaceutical policies in the CEECs are provided in Table 5 and an additional table [see Additional file 1]. Key features are as follows: Health and pharmaceutical expenditure, primarily funded by social health insurance contributions, was low compared to the Western European countries, and the share of health expenditure spent on medicines was rather high. In 2008, the EU-15 (i.e. 15 countries from Western, Northern and Southern Europe that joined the EU before 2004), spent an average of € 424 on medicines, corresponding to around 15.6 % of total health expenditure, whereas average figures equalled to € 180 and 24.4 % in the CEE-10 (the eight surveyed countries plus Estonia and Lithuania) [49, 52]. Within the eight surveyed CEECs, major discrepancies existed both in terms of the share of health expenditure spent on medicines and per capita pharmaceutical expenditure. Pharmaceutical expenditure was particularly low in Romania and Bulgaria, and also in Latvia and Poland. High increases in pharmaceutical expenditure were observed in Latvia (67 % during the three years before the survey), Romania (59 %) and Slovakia (57 %).

Private funding of health care, particularly of medicines (on average 54 % in the CEE-10), was high compared to the EU-15 with 38 % [49, 52], and there was considerable variation between the surveyed CEECs: Bulgaria had the highest share of private pharmaceutical spending (82 %), followed by Latvia (62 %) and Poland (61 %). Apart from Latvia and Poland, the share of private pharmaceutical expenditure had grown in the three years before the EHIS survey, particularly in the Czech Republic (57 %) and Hungary (38 %). Private pharmaceutical expenditure resulted from full out-of-pocket payments in the case of self-medication (non-prescribed, non-reimbursable medicines) and from co-payments of reimbursable medicines (i.e. medicines whose cost were, at least, partially covered by public payers). Market shares for non-prescription and self-medication medicines were higher than in Western European countries [53–55]. Whereas no co-payments were applied in the in-patient sector in the CEECs, they were charged for out-patient medicines: Some countries had prescription fees, and all CEECs applied co-payments as percentage of the price for those medicines that displayed a lower therapeutic value. De-listings (i.e. exclusion of medicines from reimbursement) were reported for some CEECs, and they contributed to increased private pharmaceutical expenditure.

The number of authorized medicines and of medicines on the market was usually lower in the CEECs than in the other European countries (for data on Europe see [9]). Except for Slovenia, pharmacy density was higher in the surveyed CEECs than in Western European countries (an average of 3,360 and 5,780 inhabitants per pharmacy in the EU-10 and the EU-15 respectively in 2005, [9]). But major intra-country discrepancies resulting in low accessibility to pharmacies in the rural areas existed in some CEECs (Bulgaria, Czech Republic, Poland, Romania). The sale of (specific) OTC medicines outside pharmacies was permitted in all surveyed CEECs but Latvia, Poland and Slovakia.

Pricing and reimbursement is a national competence in the EU Member States under the condition that overall procedural provisions, as laid down in the EU Transparency Directive [56] such as deadlines for pricing and reimbursement decisions, for instance, are complied with. The CEECs adapted their pricing and reimbursement systems to the ‘acquis communitaire’ before acceding to the EU in 2004 and 2007, respectively [57]. Some CEECs (Czech Republic, Slovenia, Slovakia) undertook additional changes in the years before the EHIS survey, to further strengthen their institutional framework and/or to react to new challenges, such as high-cost medicines [see country profiles in Additional file 1]. At the time of the survey, Poland was fundamentally changing its pharmaceutical pricing and reimbursement system following a European Commission’s infringement procedure related to the EU Transparency Directive that also addressed long decision times. Related to demand-side measures, measures to encourage rational prescribing and use of medicines, including a promotion of generics’ uptake, were in place in the CEECs, but limited enforcement was reported.

Overall, the literature review identified several key challenges and shortcomings in the pharmaceutical systems of the investigated CEECs: In all countries but Slovenia high co-payments and out-of-pocket payments as well a limited enforcement of measures to promote a more rational prescribing and use of medicines (including encouraging generics uptake) were an issue. Underfunding of the pharmaceutical system was reported from Bulgaria, the Czech Republic, Latvia and Romania, and informal payments on a major scale were identified in Bulgaria, Hungary, Latvia, Poland, and Romania. Bulgaria, Poland and Romania were criticized for inefficiencies in procedures and policy coordination. Slovenia was the only country out of the eight CEECs for which the above-mentioned deficits were not reported. However, Slovenia was struggling with limited availability of medicines, and so did Bulgaria, Latvia and Romania.

## Discussion

According to the analysis of EHIS survey data higher socioeconomic groups were found to have a comparably higher likelihood to use non-prescribed medicines in all eight surveyed CEECs and to use prescribed medicines in three countries (Latvia, Poland, and Romania). Our findings are partly in line with the very few studies on this issue: Geckova et al. [19] found a pro-rich socioeconomic gradient in non-prescribed medicine use and a pro-poor gradient in prescribed medicine use of adolescents in Slovakia in 1998. Gorecka et al. [20] identified geographic variation in the use of cardiovascular medicines level in the Czech Republic for the years 1997-2000 and attributed this to socioeconomic factors.

The eight CEECs have a social health insurance system in place that should, in principle, ensure coverage of the entire population and would allow linking health related social exclusion to the social policy context which is considered important [57]. The identified socioeconomic inequalities, particularly to prescribed medicine use, might result from deficits in the social insurance systems (issues of non-participation were reported for some CEECs (e.g. Bulgaria: [58]) and from lack of or inefficiencies in specific pharmaceutical policies. In the following, we explore potential factors that are able to contribute to the socioeconomic (in)equalities observed.

### Sustainable funding

A major prerequisite for equitable access to medicines is sustainable funding [38, 42]. The comparably low pharmaceutical expenditure in Romania (€ 92 per capita) and Bulgaria (€ 110 per capita), but also Latvia and Poland (around € 135 per capita) points to possible underfunding. Insufficient investment in the field of medicines could be one explanation for the socioeconomic gradient in prescribed medicine use observed in Latvia, Poland and Romania. The more than 50 % increase in per capita pharmaceutical expenditure in Latvia, Romania, Slovakia and Bulgaria in the three years before the survey could be interpreted as countries’ efforts to invest into medicines, but it might also be an indicator that the countries spent more than their economic situation allowed and thus negatively impacted sustainability.

### Affordability

Apart from Slovakia, all above mentioned countries had shares of private pharmaceutical expenditure of more than 55 % (Bulgaria as high as 82 %). The high patients’ contributions in Latvia, Romania and Poland are likely a major reason for the non-use of non-prescribed, privately funded medicines, but they might also explain the socioeconomic gradient in prescribed medicine use observed in these countries since patients were frequently required to co-pay for out-patient prescribed medicines. Further co-payments in health care could be additional barriers for patients with a lower socioeconomic background to visit a doctor and get a prescription: In the CEECs co-payments for attending a physician were, generally, in place, supplemented by ‘under-the-table payments’.

Overall economic developments are key drivers for policy-makers to curb publicly financed expenditure, even more with the global financial crisis. The Czech Republic, for instance, introduced co-payments to doctors’ visits and significantly increased the value-added tax (VAT) on medicines in 2008, and the share of the private pharmaceutical spending had grown by 57 % within three years. This could explain the socioeconomic gradient in non-prescribed use, and it might also lead to socioeconomic inequalities in prescribed medicine use though this was not confirmed by the EHIS results on the Czech Republic.

Within the CEECs Latvia, Hungary and Romania were particularly impacted by the global financial crisis [59]. Latvia responded through several cost-containment measures [60, 61]. This probably aggravated existing socioeconomic inequalities whose existence in health care (not medicines) had been identified as extensive in Latvia, also in comparison to the other Baltic countries [62]. In Romania, accessibility to prescribed, reimbursable medicines was strongly limited at the time of the survey due to high co-payments for out-patient medicines, and particularly due to the existence of pharmaceutical budgets for pharmacies: Once a monthly financial threshold had been reached a pharmacy was no longer permitted to fill prescriptions [63]. Thus, patients could not access prescribed medication unless they were willing, and able, to pay for it out-of-pocket. In 2007, after years of galloping growth rates in pharmaceutical expenditure, Hungary launched a reform to drastically curb public pharmaceutical expenditure. As a result, the share of private pharmaceutical expenditure (52 %) was rather high at the time of the survey (2009), and it had considerably grown (by 38 %) within the previous three years. This may cause concerns about limited affordability for lower socioeconomic groups [28]. Despite this austere cost-containment strategy no socioeconomic gradient in prescribed medicine use was found. A possible explanation is provided by a study on Hungary [64] according to which affordability of medicines was not negatively impacted by the cost-containment measures. It was argued that the policies were designed in a way to particularly target market participants (industry, distribution actors) rather than private households. Medicine prices were drastically cut, for instance, but this resulted in lower (percentage) co-payments for the patients.

Medicine prices should be affordable for those who pay for them – public payers and private households. There is lack of knowledge about prices of OTC medicines in general [65], and these prices have not been surveyed in the CEECs either. The socioeconomic gradient in non-prescribed medicine use that was found in all eight CEECs potentially implies that patients with a lower socioeconomic background considered medicines, which had to be paid out-of-pocket, as not affordable. In this context, attention should be paid to the value-added tax (VAT) on medicines. In many low- and middle-income countries world-wide, taxes and duties account for a considerable share of the medicine prices, thus contributing to increasing medicine prices up to an unaffordable level for the majority of the population [66]. In the CEECs, the only relevant tax related to the price of a medicine is the value-added tax. In the CEECs the VAT rates on medicines (amounting to 10 % or less) tended to be lower than the standard VAT rates [9, 67] and lower compared to the other EU countries. Nonetheless, they had risen in some countries (Czech Republic, Poland, Romania, Slovenia) from usually 5 % in 2004 [7] to their current rates of between 7.5 and 10 % since an increase in VAT rates was a common cost-containment policy [60]. While it might not appear high, it might still result in a medicine price of non-funded medicines that lower socioeconomic groups were not able, or not willing, to afford.

Little can be said about whether medicine prices were affordable for public payers in the CEECs given the lack of price studies in this region. One study [68] on 23 European countries that also included six CEECs showed that the pharmacy retail price (i.e. price at pharmacy) in the CEECs, except for Slovenia, was considerably lower than in the other European countries. However, unpublished analyses from the Pharma Price Information service of the Austrian Public Health Institute [69] indicated that at the time of the EHIS survey prices for specific new, high-cost medicines (e.g. cancer medicines, orphan medicines) were comparably high. These medicines were typically fully funded by public payers, and this ensured access to higher-priced medicines for the patients. This might explain why a socioeconomic gradient for prescribed medicine use was found in only three of the eight CEECs included in our study.

### Availability and accessibility

Limited medicine availability was an issue in the CEECs. However, availability deficits were apparently not linked to the socioeconomic background of the patients, but targeted the whole population, as in the case of Slovenia and Latvia with a low number of medicines authorized and/or on the market. These countries are considered as ‘small markets’ that might not be sufficiently attractive for the pharmaceutical industry to be supplied [70]. Limited availability also resulted from medicine shortages, i.e. that medicines were not supplied in sufficient quantity to pharmacies and hospitals. This was reported to be a major problem in Romania [71]. Furthermore, we found indications of regional inequalities: Despite the generally high pharmacy density in the CEECs (except for Slovenia), major intra-country discrepancies existed in some CEECs (Bulgaria, Czech Republic, Poland, Romania) and resulted in low accessibility to pharmacies in the rural areas. Regarding the regional variation of OTC retailers, based on the experience from Western European countries about a clustering of these dispensaries in towns with already good accessibility [65], it can be assumed that the easier access to OTC medication was likely to primarily serve well-off people in the urban areas who were willing and able to pay for these non-funded medicines. This would contribute to regional inequalities within countries, but depending on the regional distribution of socioeconomic groups can also contribute to the socioeconomic gradient identified in this paper.

### Rational selection and use of medicines

A major need for improving the pricing and reimbursement procedure was reported from Bulgaria and Romania whose processes were considered unclear and intransparent. The design of a pricing and reimbursement system is not simply a technical issue but it can considerably impact accessibility and affordability: Unclear and intransparent processes encourage corruption and an irrational selection of medicines into reimbursement, and this, eventually, can limit sustainable public funding. Cumbersome long processes, as observed for Poland, might result in delays in access to needed medicines for patients.

The CEECs have been criticized for not paying enough attention to promoting a rational use of medicines [57] that aims to ensure that patients receive the appropriate medicine at the right dose and at the time they require. The instruments to encourage rational prescribing existed in the CEECs but were apparently not fully enforced. Knowing this, however, we cannot draw conclusions as to whether the medicine use reported in the EHIS survey was appropriate, or not.

Given the limits in funding by both public payers and private households, promoting the uptake of generics is considered as a recommended policy option [72]. The CEECs already had comparably high generic market shares, since local generic industries played a strong role historically, even before the transition process [73], and the use of prescribed generics was promoted by tools such as INN prescribing and generic substitution. Still, high reluctance versus the quality of generics was reported [12]. Thus, awareness-raising campaigns to inform about generics could be a policy to reduce in the observed socioeconomic gradient in non-prescribed use, by motivating people to use lower priced generics.

### Reducing socioeconomic inequalities through pharmaceutical policies?

Summarizing, the policy analysis identified some factors that help explain the observed socioeconomic gradient in non-prescribed medicine use in the eight CEECs and in prescribed medicine use in three of them. These include a high and increasing share of private funding such as co-payments for prescribed out-patient medicines and co-payments beyond medicines (e.g. for attending doctors to get a prescription), limited investment in medicines, and long and intransparent processes regarding the inclusion of medicines into reimbursement. Further deficits such as limited availability of medicines, irrational prescribing and regional variation in the accessibility of pharmacies and OTC retailers are additional factors limiting an equitable access but it could not be determined whether, or not, lower socioeconomic groups are particularly affected. The analysis of the pharmaceutical policies in Latvia, Poland and Romania provides an indications that actually these policies contribute to the observed socioeconomic inequalities in prescribed medicine use in these countries. While the three countries cannot be seen as a very homogenous cluster in terms of pharmaceutical policies, they share an accumulation of policies that do not adequately address inequalities in medicines use. Similarly, for Bulgaria, the policy framework observed would suggest a socioeconomic gradient in prescribed medicine use. This, however, was not confirmed based on the EHIS data. Also, it might have been possible that cost-containment policies would have had a negative impact on equitable access to medicines in the Czech Republic and in Hungary. In this respect, note that the only two other studies [19, 20] on socioeconomic determinants in medicine use in CEECs, focussing on adolescents in Slovakia and on cardiovascular medicines in Czech Regions did identify a socioeconomic gradient. Though pharmaceutical policies were apparently successful in reducing socioeconomic inequalities in medicine use in some CEECs, further factors were likely to have an impact. These might be factors at the individual level of the patients (e.g. adherence to medicines) as well as overall political and economic factors, such as the stability of a health care system, patients’ trust into it and the impact of the transition processes as observed in the CEECs in the 1990-ties. Finally, the non-existence of a socioeconomic gradient in medicine use can still imply any under-use, over-use or misuse of medicines in general, and overall limited availability and accessibility of medicines for the patients of that country.

### Limitations

This study does not come without limitations. First, the quantitative analysis is based on self-reported information and hence comes with a risk of measurement errors. However, at the individual country level, where data was collected, several quality control steps were taken [74] and data comparability was also ensured by Eurostat, based e.g. on a standard questionnaire, conceptual guidelines and rationales as well as a standard translation protocol [75, 76]. Data was collected either via a stand-alone survey or as part of another (health or non-health) survey (i.e. national health interview survey, labour force survey, other household survey) based on face-to-face interviews, telephone interviews, self-administered questionnaires (or a combination of these means) [77]. Consistency and integrity checks on the national EHIS data were carried out by Eurostat, and quality reports were also disseminated [74]. Also, household surveys are an acknowledged standard tool in pharmaceutical consumption research according to the WHO [78]. Second, the EHIS household surveys goes beyond the regulatory definition of medicines in national legislations since ‘dietary supplements such as herbal medicines or vitamins’ were part of a question on medicine use (Table 3). To find out if this influences our results, sensitivity analyses were carried out excluding both the use of ‘vitamins, minerals or tonics’ or/and ‘some other type or medicine or supplement’, respectively. While the socioeconomic gradient was generally found to be less pronounced but still statistically significant in these sensitivity analyses, the qualitative conclusions regarding the socioeconomic gradient identified in the main analysis remained the same. Third, interviewees were asked about their use of ‘medicines prescribed or recommended by a doctor’ and ‘medicines not prescribed or recommended by a doctor’ (Table 3). Since we had no information about medicines recommended but not prescribed, we interpreted these two categories of ‘prescribed’ and ‘non-prescribed use’. We based this approach on the assumption that doctors tend to believe that patients expect a prescription [79]. Fourth, the analysis refers to different time periods between 2007 and 2009. We investigated the pharmaceutical policy framework at the time of the EHIS survey at national level, but for comparing national data (e.g. expenditure) versus a European or CEE average a specific year (2008) was taken as reference. This was, in some cases, not the year of the survey. Also, the survey comes with different national sample sizes. This seems especially relevant for Poland, where the analysis is based on a comparatively large study population, and thus even small differences between socioeconomic groups may become statistically significant. Finally, non-use of medicines can have numerous causes. We explored whether the pharmaceutical policy framework impacts, positively or negatively, on socioeconomic (in)equalities. However, as the wealth of literature on adherence rightly points out, there are several other reasons for not using medicines or stopping prescribed medication prematurely. To identify the relative importance of the various factors as co-determinants of the use or non-use of medicines, in-depth investigation for single countries is needed in future research.

## Conclusion

Our study confirmed a socioeconomic gradient in medicine use in Central and Eastern Europe: Socioeconomic inequalities in non-prescribed medicine use were found in all eight surveyed CEECs, whereas for prescribed medicines this was only the case in three countries (Latvia, Poland, and Romania). The latter could be an indication that pharmaceutical policies, which typically refer to publicly (co-)funded medicines, had been able to successfully address inequalities in the other CEECs. Increasing public funding for medicines, and reductions in and exemptions from co-payments for vulnerable groups are likely appropriate strategies to ensure more equitable access to medicines. In addition, an increased generics uptake, a more rational selection of medicines into reimbursement and more rational prescribing can contribute to raise resources for public spending.

Our study confirms that pharmaceutical policies, and their implementation and enforcement, are important tools to address socioeconomic inequalities. As a more general conclusion, with improved knowledge about the extent and the character of socioeconomic inequalities in medicine consumption in CEECs as well as in other parts of the world, pharmaceutical policies could be used more effectively as a major tool to improve access to medicines across entire populations.

This study refers to the years 2007-2009. Meanwhile, the CEEC region has been severely affected by the global financial crisis. The economic situation has led to increasing poverty and growing financial pressure for large parts of the populations, which, not least, can lead to changes in medicine consumption. Further, public budgets came under increasing pressure, a pressure that also tightens financial scope for pharmaceutical policies. For future research, it is suggested to follow up this study by analysing pharmaceutical policies in the CEECs during the crisis and exploring their impact on medicine use.

## Additional files

Additional file 1: Table S1. Prescribed medicine use, non-prescribed medicine use, medicine use of both types versus no medicine use; description of data: Full results for multinomial logistic regression analyses for eight individual countries. (DOCX 47 kb) Additional file 2: Table S2. Background information on pharmaceutical policies in 8 Central and Eastern European countries; description of data: Country background information giving an overview of pharmaceutical policies as well as key challenges by country. Sources: All: [49]; Bulgaria: [7, 10, 58, 83, 101, 118]; Czech Republic: [7, 9, 10, 82, 100, 109, 110, 113, 120, 121]; Hungary: [10, 12, 16, 21, 64, 85, 91, 104, 106, 119, 121]; Latvia: [60, 86, 88, 102, 107, 114, 122]; Poland: [8, 95, 96, 103, 111, 115, 123, 124]; Romania: [9, 10, 63, 71, 84, 89, 92, 94, 97, 98, 125]; Slovenia: [7, 9, 10, 70, 81, 126, 127]; Slovakia: [85, 100, 105, 108, 116, 128,129]. (DOCX 30 kb)

## Abbreviations

(T)HE: (total) health care expenditure
(T)PE: (total) pharmaceutical expenditure
BG: Bulgaria
CEE(Cs): Central and Eastern European (countries)
CI: Confidence interval
CZ: Czech Republic
D: Deductible
EHIS: European Health Interview Survey
EU: European Union
GPs: General practitioners
HC system: Health care system
HU: Hungary
INN: International Non-Proprietary Name
ISCED: International Standard Classification of Education
LV: Latvia
OECD: Organisation for Economic Co-operation and Development
OTC: Over-the-Counter medicines
PE: Pharmaceutical expenditure
PF: Prescription fee
PL: Poland
PP: Percentage points
PPRI: Pharmaceutical Pricing and Reimbursement Information
R: Reimbursement
RO: Romania
RRR: Relative-risk ratio
SI: Slovenia
SK: Slovakia
VAT: Value-added tax
WHO: World Health Organization
