# Supplementary material for: Inequalities in medicine use in Central Eastern Europe: an empirical investigation of socioeconomic determinants in eight countries
Source: Int J Equity Health. 2015 Nov 5;14:124. doi: 10.1186/s12939-015-0261-0 (PMC4635528; doi:10.1186/s12939-015-0261-0)
Supplement: Additional file 1: Table S1. — Prescribed medicine use, non-prescribed medicine use, medicine use of both types versus no medicine use; description of data: Full results for multinomial logistic regression analyses for eight individual countries. (DOCX 47 kb) [file 12939_2015_261_MOESM1_ESM.docx]

**Additional Table S1: Prescribed medicine use, non-prescribed medicine use, medicine use of both types versus no medicine use**

|  | **n** | **RRR (95% CI)** |  | **n** | **RRR (95% CI)** |  | **n** | **RRR (95% CI)** |  |
| --- | --- | --- | --- | --- | --- | --- | --- | --- | --- |
| **BG (n = 4,777)** | **Non-prescribed only** | | | **Prescribed only** | | | **Both** | | |
| **Employment status** |  |  |  |  |  |  |  |  |  |
| Employed | 499 | 1.00 |  | 352 | 1.00 |  | 194 | 1.00 |  |
| Retired | 150 | 0.70 (0.47-1.04) |  | 835 | 1.15 (0.80-1.66) |  | 383 | 1.14 (0.75-1.73) |  |
| Unemployed, disabled | 61 | 0.58 (0.42-0.81) | *** | 137 | 0.83 (0.59-1.17) |  | 60 | 0.78 (0.52-1.18) |  |
| In training, at home | *20-49* | *0.82 (0.54-1.25)* |  | *20-49* | *1.36 (0.74-2.48)* |  | *<20* | *-* |  |
| **Highest education** |  |  |  |  |  |  |  |  |  |
| ISCED ≤ 2 | 190 | 1.00 |  | 603 | 1.00 |  | 229 | 1.00 |  |
| ISCED 3-4 | 411 | 1.05 (0.84-1.31) |  | 568 | 1.09 (0.87-1.37) |  | 310 | 1.53 (1.17-1.99) | ** |
| ISCED 5-6 | 146 | 1.05 (0.79-1.40) |  | 178 | 1.32 (0.97-1.81) |  | 113 | 2.13 (1.51-3.03) | *** |
| **Income quintile** |  |  |  |  |  |  |  |  |  |
| 1 | 131 | 1.00 |  | 539 | 1.00 |  | 239 | 1.00 |  |
| 2 | 59 | 0.87 (0.60-1.25) |  | 193 | 0.91 (0.66-1.25) |  | 84 | 0.86 (0.59-1.24) |  |
| 3 | 107 | 1.03 (0.74-1.41) |  | 189 | 0.93 (0.68-1.27) |  | 105 | 1.08 (0.76-1.54) |  |
| 4-5 | 450 | 1.23 (0.93-1.63) |  | 428 | 0.88 (0.67-1.16) |  | 224 | 0.88 (0.64-1.20) |  |
| **Age** |  |  |  |  |  |  |  |  |  |
| 18-29 | 101 | 1.00 |  | 40 | 1.00 |  | 26 | 1.00 |  |
| 30-39 | 144 | 1.38 (1.01-1.88) | * | *20-49* | *1.09 (0.65-1.83)* |  | *20-49* | *1.14 (0.63-2.08)* |  |
| 40-49 | 159 | 1.52 (1.11-2.08) | ** | 130 | 2.08 (1.30-3.31) | ** | 64 | 1.63 (0.93-2.83) |  |
| 50-59 | 172 | 1.79 (1.31-2.44) | *** | 254 | 2.73 (1.75-4.26) | *** | 129 | 2.29 (1.36-3.87) | ** |
| 60-69 | 103 | 2.13 (1.37-3.32) | *** | 366 | 3.70 (2.22-6.18) | *** | 184 | 3.27 (1.79-5.95) | *** |
| 70+ | 68 | 2.03 (1.19-3.47) | ** | 511 | 4.94 (2.83-8.60) | *** | 216 | 4.00 (2.09-7.64) | *** |
| **Gender** |  |  |  |  |  |  |  |  |  |
| Male | 301 | 1.00 |  | 591 | 1.00 |  | 208 | 1.00 |  |
| Female | 446 | 1.84 (1.55-2.19) | *** | 758 | 1.31 (1.08-1.58) | ** | 444 | 2.26 (1.81-2.82) | *** |
| **Chronic conditions** |  |  |  |  |  |  |  |  |  |
| No | 588 | 1.00 |  | 167 | 1.00 |  | 99 | 1.00 |  |
| Yes | 159 | 1.37 (1.07-1.76) | ** | 1,182 | 16.56 (13.16-20.84) | *** | 553 | 15.40 (11.61-20.44) | *** |
| **Self-assessed health** |  |  |  |  |  |  |  |  |  |
| Good | 603 | 1.00 |  | 368 | 1.00 |  | 216 | 1.00 |  |
| Bad | 144 | 1.04 (0.79-1.35) |  | 981 | 2.20 (1.75-2.76) | *** | 436 | 1.87 (1.44-2.44) | *** |
| **Constant** |  | 0.16 (0.11-0.24) | *** |  | 0.03 (0.02-0.06) | *** |  | 0.01 (0.01-0.02) | *** |
| McFadden’s R²: 0.239 |  |  |  |  |  |  |  |  |  |
| **CZ (n = 1.454)** | **Non-prescribed only** | | | **Prescribed only** | | | **Both** | | |
| **Employment status** |  |  |  |  |  |  |  |  |  |
| Employed | 211 | 1.00 |  | 148 | 1.00 |  | 143 | 1.00 |  |
| Retired | *20-49* | *0.54 (0.22-1.28)* |  | 222 | 1.03 (0.51-2.07) |  | 186 | 1.42 (0.68-2.97) |  |
| Unemployed, disabled | *<20* | *-* |  | *20-49* | *1.38 (0.66-2.88)* |  | *20* | *0.88 (0.40-1.93)* |  |
| In training, at home | *20-49* | *1.15 (0.66-2.00)* |  | *<20* | *-* |  | *20-49* | *1.76 (0.91-3.39)* |  |
| **Highest education** |  |  |  |  |  |  |  |  |  |
| ISCED ≤ 2 | 20 | 1.00 |  | 100 | 1.00 |  | 57 | 1.00 |  |
| ISCED 3-4 | 221 | 1.93 (1.08-3.46) | ** | 289 | 0.91 (0.56-1.48) |  | 287 | 1.69 (1.01-2.85) | * |
| ISCED 5-6 | 50 | 3.08 (1.51-6.26) | ** | *20-49* | *0.80 (0.39-1.64)* |  | *20-49* | *1.47 (0.70-3.05)* |  |
| **Income quintile** |  |  |  |  |  |  |  |  |  |
| 1 | 13 | 1.00 |  | 82 | 1.00 |  | 61 | 1.00 |  |
| 2 | *20-49* | *2.04 (0.90-4.62)* |  | 74 | 1.31 (0.67-2.55) |  | 67 | 1.57 (0.79-3.12) |  |
| 3 | 63 | 1.95 (0.91-4.22) |  | 129 | 1.50 (0.82-2.77) |  | 107 | 1.55 (0.82-2.92) |  |
| 4-5 | 181 | 1.96 (0.92-4.17) |  | 136 | 1.42 (0.76-2.65) |  | 141 | 1.56 (0.82-2.97) |  |
| **Age** |  |  |  |  |  |  |  |  |  |
| 18-29 | 79 | 1.00 |  | 27 | 1.00 |  | 38 | 1.00 |  |
| 30-39 | 80 | 0.88 (0.57-1.37) |  | *20-49* | *1.27 (0.67-2.42)* |  | *20-49* | 1.23 (0.68-2.22) |  |
| 40-49 | 51 | 1.05 (0.62-1.75) |  | *20-49* | *2.39 (1.22-4.69)* | ** | *20-49* | 2.18 (1.66-4.10) | * |
| 50-59 | *20-49* | *1.22 (0.71-2.11)* |  | 90 | 4.53 (2.35-8.73) | *** | 57 | 1.96 (2.62-3.76) | * |
| 60-69 | *20-49* | *0.98 (0.41-2.32)* |  | 110 | 7.67 (3.31-17.76) | *** | 100 | 3.94 (3.68-9.23) | ** |
| 70+ | *<20* | *-* |  | 123 | 18.55 (6.47-53.18) | *** | 88 | 7.12 (6.64-20.75) | *** |
| **Gender** |  |  |  |  |  |  |  |  |  |
| Male | 136 | 1.00 |  | 198 | 1.00 |  | 144 | 1.00 |  |
| Female | 155 | 1.63 (1.18-2.26) | ** | 223 | 1.35 (0.96-1.90) |  | 232 | 2.10 (1.49-2.98) | *** |
| **Chronic conditions** |  |  |  |  |  |  |  |  |  |
| No | 218 | 1.00 |  | 113 | 1.00 |  | 94 | 1.00 |  |
| Yes | 73 | 1.50 (1.01-2.25) | * | 308 | 6.91 (4.73-10.10) | *** | 282 | 8.98 (6.10-13.23) | *** |
| **Self-assessed health** |  |  |  |  |  |  |  |  |  |
| Good | 237 | 1.00 |  | 185 | 1.00 |  | 177 | 1.00 |  |
| Bad | 54 | 0.92 (0.59-1.43) |  | 236 | 1.38 (0.92-2.08) |  | 199 | 1.47 (0.97-2.23) |  |
| **Constant** |  | 0.16 (0.06-0.43) | *** |  | 0.08 (0.03-0.20) | *** |  | 0.04 (0.02-0.10) | *** |
| McFadden’s R²: 0.171 |  |  |  |  |  |  |  |  |  |
| **HU (n = 4,479)** | **Non-prescribed only** | | | **Prescribed only** | | | **Both** | | |
| **Employment status** |  |  |  |  |  |  |  |  |  |
| Employed | 486 | 1.00 |  | 465 | 1.00 |  | 380 | 1.00 |  |
| Retired | 56 | 1.05 (0.58-1.90) |  | 644 | 0.96 (0.62-1.51) |  | 370 | 0.84 (0.52-1.35) |  |
| Unemployed, disabled | 59 | 0.81 (0.57-1.14) |  | 270 | 1.13 (0.85-1.50) |  | 165 | 1.14 (0.84-1.56) |  |
| In training, at home | 70 | 1.67 (1.16-2.41) | ** | 70 | 1.69 (1.11-2.57) | ** | *20-49* | *1.05 (0.63-1.75)* |  |
| **Highest education** |  |  |  |  |  |  |  |  |  |
| ISCED ≤ 2 | 65 | 1.00 |  | 584 | 1.00 |  | 244 | 1.00 |  |
| ISCED 3-4 | 386 | 1.97 (1.44-2.70) | *** | 721 | 0.96 (0.76-1.22) |  | 492 | 1.54 (1.18-2.01) | ** |
| ISCED 5-6 | 220 | 4.32 (2.98-6.27) | *** | 144 | 1.01 (0.71-1.41) |  | 208 | 3.19 (2.24-4.55) | *** |
| **Income quintile** |  |  |  |  |  |  |  |  |  |
| 1 | 76 | 1.00 |  | 485 | 1.00 |  | 208 | 1.00 |  |
| 2 | 92 | 1.06 (0.74-1.52) |  | 320 | 1.04 (0.78-1.37) |  | 199 | 1.43 (0.54-1.96) | * |
| 3 | 158 | 1.40 (1.00-1.96) |  | 287 | 0.94 (0.71-1.24) |  | 216 | 1.51 (1.10-2.06) | * |
| 4-5 | 345 | 1.66 (1.20-2.31) | ** | 357 | 1.11 (0.84-1.47) |  | 321 | 1.88 (1.38-2.56) | *** |
| **Age** |  |  |  |  |  |  |  |  |  |
| 18-29 | 167 | 1.00 |  | 96 | 1.00 |  | 72 | 1.00 |  |
| 30-39 | 225 | 1.30 (0.98-1.72) |  | 129 | 1.11 (0.78-1.59) |  | 112 | 0.94 (0.64-1.39) |  |
| 40-49 | 139 | 1.17 (0.85-1.60) |  | 176 | 1.48 (1.03-2.12) | ** | 103 | 0.85 (0.57-1.28) |  |
| 50-59 | 84 | 0.76 (0.53-1.08) |  | 325 | 2.21 (1.56-3.15) | *** | 236 | 1.65 (1.12-2.42) | * |
| 60-69 | *20-49* | *0.91 (0.48-1.74)* |  | 330 | 4.40 (2.63-7.38) | *** | 207 | 3.13 (1.81-5.43) | *** |
| 70+ | *<20* | *-* | * | 393 | 6.52 (3.65-11.67) | *** | 214 | 4.84 (2.60-9.01) | *** |
| **Gender** |  |  |  |  |  |  |  |  |  |
| Male | 291 | 1.00 |  | 607 | 1.00 |  | 329 | 1.00 |  |
| Female | 380 | 1.62 (1.33-1.96) | *** | 842 | 1.43 (1.18-1.73) | *** | 615 | 2.23 (1.81-2.74) | *** |
| **Chronic conditions** |  |  |  |  |  |  |  |  |  |
| No | 288 | 1.00 |  | 63 | 1.00 |  | 37 | 1.00 |  |
| Yes | 383 | 2.84 (2.29-3.52) | *** | 1,386 | 17.79 (13.29-23.82) | *** | 907 | 21.91 (15.21-31.55) | *** |
| **Health status** |  |  |  |  |  |  |  |  |  |
| Good | 522 | 1.00 |  | 407 | 1.00 |  | 273 | 1.00 |  |
| Bad | 149 | 0.95 (0.73-1.23) |  | 1,042 | 2.03 (1.63-2.53) | *** | 671 | 2.85 (2.23-3.63) | *** |
| **Constant** |  | 0.07 (0.05-0.12) | *** |  | 0.03 (0.02-0.05) | *** |  | 0.01 (0.00-0.01) | *** |
| McFadden’s R²: 0.223 |  |  |  |  |  |  |  |  |  |
| **LV (n = 5,920)** | **Non-prescribed only** | | | **Prescribed only** | | | **Both** | | |
| **Employment status** |  |  |  |  |  |  |  |  |  |
| Employed | 852 | 1.00 |  | 545 | 1.00 |  | 487 | 1.00 |  |
| Retired | 161 | 0.75 (0.50-1.11) |  | 636 | 0.96 (0.66-1.38) |  | 462 | 1.06 (0.72-1.56) |  |
| Unemployed, disabled | 118 | 0.87 (0.66-1.13) |  | 134 | 1.01 (0.75-1.36) |  | 81 | 0.77 (0.55-1.08) |  |
| In training, at home | 174 | 0.96 (0.76-1.21) |  | 65 | 0.74 (0.52-1.04) |  | 52 | 0.65 (0.45-0.94) | * |
| **Highest education** |  |  |  |  |  |  |  |  |  |
| ISCED ≤ 2 | 281 | 1.00 |  | 495 | 1.00 |  | 287 | 1.00 |  |
| ISCED 3-4 | 680 | 1.23 (1.03-1.48) | ** | 637 | 1.18 (0.97-1.45) |  | 564 | 1.70 (1.36-2.12) | *** |
| ISCED 5-6 | 344 | 2.07 (1.64-2.62) | *** | 248 | 2.09 (1.59-2.73) | *** | 132 | 3.00 (2.25-4.01) | *** |
| **Income quintile** |  |  |  |  |  |  |  |  |  |
| 1 | 48 | 1.00 |  | 35 | 1.00 |  | 27 | 1.00 |  |
| 2 | 346 | 0.94 (0.63-1.39) |  | 635 | 1.72 (1.06-2.78) | ** | 448 | 1.46 (0.86-2.47) |  |
| 3 | 648 | 1.08 (0.73-1.60) |  | 571 | 2.10 (1.29-3.42) | ** | 472 | 1.92 (1.13-3.25) | * |
| 4-5 | 263 | 0.96 (0.63-1.46) |  | 139 | 1.99 (1.17-3.37) | * | 135 | 2.01 (1.14-3.56) | * |
| **Age** |  |  |  |  |  |  |  |  |  |
| 18-29 | 353 | 1.00 |  | 111 | 1.00 |  | 97 | 1.00 |  |
| 30-39 | 269 | 1.00 (0.81-1.24) |  | 118 | 1.05 (0.76-1.44) |  | 85 | 0.83 (0.58-1.17) |  |
| 40-49 | 265 | 1.06 (0.85-1.33) |  | 152 | 1.06 (0.77-1.46) |  | 142 | 1.04 (0.75-1.46) |  |
| 50-59 | 202 | 1.16 (0.90-1.48) |  | 252 | 2.12 (1.55-2.90) | *** | 219 | 2.09 (1.50-2.90) | *** |
| 60-69 | 122 | 1.20 (0.83-1.74) |  | 311 | 3.26 (2.18-4.86) | *** | 247 | 2.79 (1.82-4.27) | *** |
| 70+ | 94 | 1.52 (0.94-2.46) |  | 436 | 5.77 (3.60-9.24) | *** | 292 | 4.31 (2.61-7.11) | *** |
| **Gender** |  |  |  |  |  |  |  |  |  |
| Male | 550 | 1.00 |  | 477 | 1.00 |  | 292 | 1.00 |  |
| Female | 755 | 1.84 (1.59-2.13) | *** | 903 | 2.40 (2.02-2.85) | *** | 789 | 3.39 (2.82-4.09) | *** |
| **Chronic conditions** |  |  |  |  |  |  |  |  |  |
| No | 958 | 1.00 |  | 318 | 1.00 |  | 259 | 1.00 |  |
| Yes | 347 | 1.57 (1.29-1.92) | *** | 1,062 | 7.51 (6.12-9.22) | *** | 823 | 8.07 (6.47-10.07) | *** |
| **Self-assessed health** |  |  |  |  |  |  |  |  |  |
| Good | 753 | 1.00 |  | 235 | 1.00 |  | 205 | 1.00 |  |
| Bad | 522 | 1.39 (1.16-1.67) | *** | 1,145 | 2.33 (1.86-2.91) | *** | 877 | 2.23 (1.75-2.83) | *** |
| **Constant** |  | 0.27 (0.17-0.41) | *** |  | 0.02 (0.01-0.04) | *** |  | 0.01 (0.01-0.03) | *** |
| McFadden’s R²: 0.176 |  |  |  |  |  |  |  |  |  |
| **PL (n = 23,244)** | **Non-prescribed only** | | | **Prescribed only** | | | **Both** | | |
| **Employment status** |  |  |  |  |  |  |  |  |  |
| Employed | 3,281 | 1.00 |  | 1,548 | 1.00 |  | 1,906 | 1.00 |  |
| Retired | 633 | 0.79 (0.66-0.95) |  | 2,908 | 1.34 (1.12-1.60) | ** | 2,896 | 1.21 (1.01-1.45) | * |
| Unemployed, disabled | 477 | 0.72 (0.63-0.82) | *** | 843 | 1.24 (1.07-1.44) | ** | 772 | 1.05 (0.90-1.21) |  |
| In training, at home | 910 | 1.12 (1.00-1.27) |  | 457 | 1.29 (1.10-1.52) | ** | 536 | 1.26 (1.08-1.48) | ** |
| **Highest education** |  |  |  |  |  |  |  |  |  |
| ISCED ≤ 2 | 815 | 1.00 |  | 2,086 | 1.00 |  | 1,790 | 1.00 |  |
| ISCED 3-4 | 3,452 | 1.34 (1.20-1.49) | *** | 3,082 | 1.32 (1.17-1.48) | *** | 3,356 | 1.68 (1.49-1.89) | *** |
| ISCED 5-6 | 1,034 | 1.79 (1.55-2.07) | *** | 588 | 1.92 (1.62-2.27) | *** | 964 | 3.52 (2.99-4.15) | *** |
| **Income quintile** |  |  |  |  |  |  |  |  |  |
| 1 | 504 | 1.00 |  | 1,050 | 1.00 |  | 1,020 | 1.00 |  |
| 2 | 1,019 | 1.14 (0.99-1.32) |  | 1,473 | 1.15 (0.99-1.34) |  | 1,463 | 1.18 (1.02-1.38) | * |
| 3 | 1,124 | 1.19 (1.03-1.37) | ** | 1,338 | 1.27 (1.09-1.48) | ** | 1,360 | 1.29 (1.11-1.51) | *** |
| 4-5 | 2,654 | 1.38 (1.20-1.58) | *** | 1,895 | 1.48 (1.28-1.72) | *** | 2,267 | 1.59 (1.37-1.85) | *** |
| **Age** |  |  |  |  |  |  |  |  |  |
| 18-29 | 1,465 | 1.00 |  | 386 | 1.00 |  | 462 | 1.00 |  |
| 30-39 | 1,253 | 1.06 (0.95-1.18) |  | 392 | 1.03 (0.86-1.23) |  | 484 | 0.99 (0.84-1.17) |  |
| 40-49 | 1,066 | 1.01 (0.90-1.14) |  | 627 | 1.25 (1.05-1.48) | ** | 772 | 1.25 (1.06-1.47) | ** |
| 50-59 | 937 | 1.02 (0.90-1.15) |  | 1,356 | 1.98 (1.68-2.33) | *** | 1,468 | 1.84 (1.57-2.15) | *** |
| 60-69 | 353 | 0.97 (0.78-1.20) |  | 1,307 | 3.03 (2.42-3.78) | *** | 1,364 | 2.91 (2.33-3.63) | *** |
| 70+ | 227 | 1.18 (0.90-1.55) |  | 1,688 | 5.73 (4.43-7.42) | *** | 1,560 | 5.14 (3.97-6.65) | *** |
| **Gender** |  |  |  |  |  |  |  |  |  |
| Male | 2,117 | 1.00 |  | 2,463 | 1.00 |  | 1,991 | 1.00 |  |
| Female | 3,184 | 1.96 (1.81-2.12) | *** | 3,293 | 1.66 (1.52-1.82) | *** | 4,119 | 2.64 (2.41-2.89) | *** |
| **Chronic conditions** |  |  |  |  |  |  |  |  |  |
| No | 3,698 | 1.00 |  | 882 | 1.00 |  | 894 | 1.00 |  |
| Yes | 1,603 | 1.88 (1.70-2.08) | *** | 4,874 | 8.84 (7.90-9.90) | *** | 5,216 | 10.77 (9.62-12.05) | *** |
| **Self-assessed health** |  |  |  |  |  |  |  |  |  |
| Good | 4,105 | 1.00 |  | 1,514 | 1.00 |  | 1,743 | 1.00 |  |
| Bad | 1,196 | 1.25 (1.12-1.41) | *** | 4,242 | 2.83 (2.52-3.18) | *** | 4,367 | 2.87 (2.56-3.23) | *** |
| **Constant** |  | 0.31 (0.26-0.37) | *** |  | 0.04 (0.03-0.05) | *** |  | 0.03 (0.02-0.03) | *** |
| McFadden’s R²: 0.195 |  |  |  |  |  |  |  |  |  |
| **RO (n = 16,381)** | **Non-prescribed only** | | | **Prescribed only** | | | **Both** | | |
| **Employment status** |  |  |  |  |  |  |  |  |  |
| Employed | 692 | 1.00 |  | 1,034 | 1.00 |  | 308 | 1.00 |  |
| Retired | 245 | 0.92 (0.71-1.18) |  | 3,143 | 1.84 (1.34-2.52) | *** | 819 | 1.67 (1.18-2.36) | ** |
| Unemployed, disabled | *20-49* | *0.81 (0.54-1.21)* |  | 248 | 0.90 (0.54-1.49) |  | 54 | 0.72 (0.41-1.28) |  |
| In training, at home | 202 | 1.01 (0.84-1.21) |  | 376 | 1.11 (0.81-1.52) |  | 111 | 1.04 (0.72-1.49) |  |
| **Highest education** |  |  |  |  |  |  |  |  |  |
| ISCED ≤ 2 | 305 | 1.00 |  | 2,470 | 1.00 |  | 623 | 1.00 |  |
| ISCED 3-4 | 714 | 1.36 (1.15-1.60) | *** | 2,054 | 1.31 (1.04-1.66) | * | 569 | 1.51 (1.17-1.96) | ** |
| ISCED 5-6 | 147 | 1.76 (1.38-2.25) | *** | 277 | 1.73 (1.19-2.54) | ** | 100 | 2.67 (1.75-4.06) | *** |
| **Income quintile** |  |  |  |  |  |  |  |  |  |
| 1 | 573 | 1.00 |  | 3,213 | 1.00 |  | 858 | 1.00 |  |
| 2 | 516 | 1.13 (0.99-1.30) |  | 1,437 | 1.33 (1.07-1.64) | ** | 393 | 1.25 (0.99-1.59) |  |
| 3 | 64 | 1.34 (0.99-1.80) |  | 115 | 0.69 (0.39-1.21) |  | *20-49* | *0.65 (0.35-1.23)* |  |
| 4-5 | *<20* | *-* |  | *20-49* | *1.13 (0.44-2.91)* |  | *<20* | *-* |  |
| **Age** |  |  |  |  |  |  |  |  |  |
| 18-29 | 214 | 1.00 |  | 171 | 1.00 |  | 41 | 1.00 |  |
| 30-39 | 252 | 1.06 (0.87-1.30) |  | 252 | 0.89 (0.62-1.29) |  | 87 | 1.24 (0.77-1.98) |  |
| 40-49 | 248 | 1.31 (1.07-1.60) | ** | 473 | 1.01 (0.70-1.45) |  | 121 | 1.09 (0.68-1.77) |  |
| 50-59 | 213 | 1.43 (1.15-1.78) | *** | 1,090 | 1.11 (0.78-1.60) |  | 314 | 1.44 (0.91-2.26) |  |
| 60-69 | 150 | 1.90 (1.39-2.59) | *** | 1,148 | 0.95 (0.61-1.49) |  | 301 | 1.17 (0.68-1.99) |  |
| 70+ | 89 | 1.49 (1.03-2.16) | * | 1,667 | 0.82 (0.51-1.33) |  | 428 | 1.02 (0.58-1.81) |  |
| **Gender** |  |  |  |  |  |  |  |  |  |
| Male | 388 | 1.00 |  | 1,977 | 1.00 |  | 432 | 1.00 |  |
| Female | 778 | 2.40 (2.10-2.74) | *** | 2,824 | 1.71 (1.41-2.08) | *** | 860 | 2.47 (1.98-3.07) | *** |
| **Chronic conditions** |  |  |  |  |  |  |  |  |  |
| No | 1,124 | 1.00 |  | 243 | 1.00 |  | 85 | 1.00 |  |
| Yes | *20-49* | *2.39 (1.64-3.47)* | *** | 4,558 | 679.32 (531.93-867.55) | *** | 1,207 | 561.24 (409.08-769.98) | *** |
| **Self-assessed health** |  |  |  |  |  |  |  |  |  |
| Good | 1,013 | 1.00 |  | 947 | 1.00 |  | 287 | 1.00 |  |
| Bad | 153 | 1.21 (0.98-1.49) |  | 3,854 | 3.09 (2.45-3.90) | *** | 1,005 | 2.92 (2.24-3.80) | *** |
| **Constant** |  | 0.04 (0.03-0.06) | *** |  | 0.01 (0.01-0.02) | *** |  | 0.00 (0.00-0.00) | *** |
| McFadden’s R²: 0.502 |  |  |  |  |  |  |  |  |  |
| **SI (n = 1,507)** | **Non-prescribed only** | | | **Prescribed only** | | | **Both** | | |
| **Employment status** |  |  |  |  |  |  |  |  |  |
| Employed | 190 | 1.00 |  | 165 | 1.00 |  | 97 | 1.00 |  |
| Retired | *20-49* | *1.26 (0.58-2.72)* |  | 213 | 0.95 (0.50-1.79) |  | 112 | 0.86 (0.42-1.77) |  |
| Unemployed, disabled | *20-49* | *1.13 (0.64-1.98)* |  | *20-49* | *0.82 (0.47-1.45)* |  | *20-49* | *0.83 (0.43-1.60)* |  |
| In training, at home | 50 | 1.46 (0.88-2.41) |  | *20-49* | *1.07 (0.60-1.93)* |  | *20-49* | *0.74 (0.37-1.47)* |  |
| **Highest education** |  |  |  |  |  |  |  |  |  |
| ISCED ≤ 2 | 75 | 1.00 |  | 266 | 1.00 |  | 122 | 1.00 |  |
| ISCED 3-4 | 186 | 1.90 (1.33-2.71) | *** | 166 | 1.11 (0.78-1.57) |  | 102 | 1.44 (0.96-2.16) |  |
| ISCED 5-6 | *20-49* | *2.27 (1.30-3.98)* | ** | *27* | *1.20 (0.64-2.26)* |  | *20-49* | *2.71 (1.39-5.26)* | ** |
| **Income quintile** |  |  |  |  |  |  |  |  |  |
| 1 | 21 | 1.00 |  | 128 | 1.00 |  | 54 | 1.00 |  |
| 2 | *20-49* | *0.97 (0.51-1.82)* |  | 122 | 0.63 (0.38-1.03) |  | 71 | 0.82 (0.46-1.45) |  |
| 3 | 57 | 1.45 (0.77-2.73) |  | 78 | 0.79 (0.46-1.37) |  | *20-49* | *0.94 (0.50-1.77)* |  |
| 4-5 | 180 | 1.89 (1.04-3.47) | * | 131 | 0.82 (0.49-1.38) |  | 82 | 1.00 (0.55-1.83) |  |
| **Age** |  |  |  |  |  |  |  |  |  |
| 18-29 | 87 | 1.00 |  | 30 | 1.00 |  | 25 | 1.00 |  |
| 30-39 | 84 | 1.39 (0.88-2.20) |  | *20-49* | *1.66 (0.90-3.04)* |  | *20-49* | *1.07 (0.54-2.12)* |  |
| 40-49 | 57 | 1.10 (0.67-1.80) |  | 71 | 2.06 (1.15-3.70) | ** | *20-49* | *0.85 (0.43-1.69)* |  |
| 50-59 | *20-49* | *1.21 (0.71-2.08)* |  | 102 | 3.55 (1.98-6.34) | *** | 60 | 2.27 (1.19-4.33) | * |
| 60-69 | *20-49* | *1.19 (0.48-2.96)* |  | 96 | 7.84 (3.57-17.25) | *** | 62 | 5.86 (2.45-14.01) | *** |
| 70+ | *<20* | *-* |  | 120 | 12.61 (5.07-31.33) | *** | *20-49* | *5.49 (2.00-15.09)* | *** |
| **Gender** |  |  |  |  |  |  |  |  |  |
| Male | 116 | 1.00 |  | 223 | 1.00 |  | 88 | 1.00 |  |
| Female | 187 | 1.98 (1.46-2.68) | *** | 236 | 1.23 (0.90-1.67) |  | 164 | 2.39 (1.66-3.43) | *** |
| **Chronic conditions** |  |  |  |  |  |  |  |  |  |
| No | 244 | 1.00 |  | 162 | 1.00 |  | 78 | 1.00 |  |
| Yes | 59 | 1.50 (1.00-2.26) |  | 297 | 5.70 (4.00-8.12) | *** | 174 | 7.79 (5.18-11.71) | *** |
| **Self-assessed health** |  |  |  |  |  |  |  |  |  |
| Good | 246 | 1.00 |  | 164 | 1.00 |  | 94 | 1.00 |  |
| Bad | 57 | 1.14 (0.75-1.73) |  | 295 | 2.49 (1.74-3.57) | *** | 158 | 2.76 (1.81-4.19) | *** |
| **Constant** |  | 0.13 (0.06-0.27) | *** |  | 0.13 (0.06-0.26) | *** |  | 0.05 (0.02-0.11) | *** |
| McFadden’s R²: 0.186 |  |  |  |  |  |  |  |  |  |
| **SK (n = 4,116)** | **Non-prescribed only** | | | **Prescribed only** | | | **Both** | | |
| **Employment status** |  |  |  |  |  |  |  |  |  |
| Employed | 614 | 1.00 |  | 404 | 1.00 |  | 464 | 1.00 |  |
| Retired | *20-49* | *0.72 (0.31-1.68)* |  | 379 | 1.11 (0.57-2.16) |  | 414 | 1.36 (0.71-2.62) |  |
| Unemployed, disabled | *20-49* | *0.66 (0.45-0.96)* | * | 114 | 1.10 (0.76-1.59) |  | 115 | 1.15 (0.80-1.65) |  |
| In training, at home | 106 | 1.20 (0.88-1.64) |  | *20-49* | *1.37 (0.86-2.17)* |  | 52 | 0.96 (0.62-1.48) |  |
| **Highest education** |  |  |  |  |  |  |  |  |  |
| ISCED ≤ 2 | 34 | 1.00 |  | 167 | 1.00 |  | 163 | 1.00 |  |
| ISCED 3-4 | 556 | 1.72 (1.12-2.63) | * | 650 | 1.17 (0.80-1.70) |  | 691 | 1.27 (0.87-1.85) |  |
| ISCED 5-6 | 220 | 2.54 (1.61-4.01) | *** | 126 | 1.11 (0.71-1.73) |  | 191 | 1.65 (1.07-2.54) | ** |
| **Income quintile** |  |  |  |  |  |  |  |  |  |
| 1 | 45 | 1.00 |  | 204 | 1.00 |  | 198 | 1.00 |  |
| 2 | 146 | 1.14 (0.75-1.75) |  | 252 | 1.02 (0.70-1.50) |  | 270 | 1.19 (0.81-1.76) |  |
| 3 | 216 | 1.33 (0.87-2.02) |  | 231 | 1.09 (0.74-1.62) |  | 245 | 1.28 (0.86-1.90) |  |
| 4-5 | 403 | 1.40 (0.92-2.11) |  | 256 | 0.99 (0.67-1.47) |  | 332 | 1.42 (0.96-2.11) |  |
| **Age** |  |  |  |  |  |  |  |  |  |
| 18-29 | 220 | 1.00 |  | 89 | 1.00 |  | 1,240 | 1.00 |  |
| 30-39 | 265 | 1.68 (1.31-2.16) | *** | 102 | 1.34 (0.91-1.98) |  | 130 | 1.09 (0.76-1.54) |  |
| 40-49 | 173 | 1.37 (1.03-1.82) | * | 131 | 1.59 (1.07-2.37) | * | 138 | 1.07 (0.74-1.55) |  |
| 50-59 | 108 | 1.30 (0.94-1.79) |  | 230 | 3.20 (2.16-4.75) | *** | 239 | 2.18 (1.51-3.15) | *** |
| 60-69 | *20-49* | *1.07 (0.46-2.50)* |  | 198 | 4.34 (2.12-8.89) | *** | 238 | 3.03 (1.50-6.10) | ** |
| 70+ | *<20* | *-* |  | 193 | 7.11 (3.02-16.73) | *** | 176 | 3.93 (1.70-9.12) | *** |
| **Gender** |  |  |  |  |  |  |  |  |  |
| Male | 378 | 1.00 |  | 455 | 1.00 |  | 351 | 1.00 |  |
| Female | 432 | 1.59 (1.33-1.91) | *** | 488 | 1.31 (1.05-1.62) | * | 694 | 2.56 (2.08-3.17) | *** |
| **Chronic conditions** |  |  |  |  |  |  |  |  |  |
| No | 516 | 1.00 |  | 72 | 1.00 |  | 97 | 1.00 |  |
| Yes | 294 | 1.62 (1.32-2.00) | *** | 871 | 17.90 (13.42-23.87) | *** | 948 | 15.13 (11.63-19.69) | *** |
| **Self-assessed health** |  |  |  |  |  |  |  |  |  |
| Good | 660 | 1.00 |  | 340 | 1.00 |  | 404 | 1.00 |  |
| Bad | 150 | 1.39 (1.05-1.82) |  | 603 | 2.16 (1.66-2.82) | *** | 641 | 2.30 (1.77-2.98) | *** |
| **Constant** |  | 0.14 (0.07-0.24) | *** |  | 0.03 (0.02-0.05) | *** |  | 0.02 (0.01-0.04) | *** |
| McFadden’s R²: 0.216 |  |  |  |  |  |  |  |  |  |

Note: Multinomial multivariate logistic regression with no medicine use as reference group; n = number of cases, RRR = multivariate relative risk ratio, CI = 95% confidence interval, *** = significant at 0.01%, ** = significant at 0.1%, * = significant at 5%. For confidentiality reasons, less than 20 observations (<20) and respective results were omitted; for low number of observations (20-49), the actual n is not reported and respective results are highlighted in italics.

Regarding the influence of health conditions on medicine consumption, in the most exceptional case, Romania, individuals with chronic conditions were attributed an almost 700 times higher chance of taking prescribed medicines. In fact, there is a close link between prescribed medicine consumption and chronic conditions also from a descriptive perspective: out of all people taking prescribed medicines in Romania, 95% are chronically ill and prescribed medicine use (in contrast to non-prescribed medicine consumption, the consumption of both prescribed and non-prescribed medicines and no medicine use) is reported by 77% of the respondents with chronic conditions.

Source: Data provided by Eurostat [43]; calculation and presentation by the authors
