# Supplementary material for: Inequalities in medicine use in Central Eastern Europe: an empirical investigation of socioeconomic determinants in eight countries
Source: Int J Equity Health. 2015 Nov 5;14:124. doi: 10.1186/s12939-015-0261-0 (PMC4635528; doi:10.1186/s12939-015-0261-0)
Supplement: Additional file 2: Table S2. — Background information on pharmaceutical policies in 8 Central and Eastern European countries; description of data: Country background information giving an overview of pharmaceutical policies as well as key challenges by country. Sources: All: [49]; Bulgaria: [7, 10, 58, 83, 101, 118]; Czech Republic: [7, 9, 10, 82, 100, 109, 110, 113, 120, 121]; Hungary: [10, 12, 16, 21, 64, 85, 91, 104, 106, 119, 121]; Latvia: [60, 86, 88, 102, 107, 114, 122]; Poland: [8, 95, 96, 103, 111, 115, 123, 124]; Romania: [9, 10, 63, 71, 84, 89, 92, 94, 97, 98, 125]; Slovenia: [7, 9, 10, 70, 81, 126, 127]; Slovakia: [85, 100, 105, 108, 116, 128,129]. (DOCX 30 kb) [file 12939_2015_261_MOESM2_ESM.docx]

**Additional Table S2: Background information on pharmaceutical policies in 8 Central and Eastern European countries**

| **Bulgaria (2008)** | **Czech Republic (2008)** |
| --- | --- |
| **Overview:**  Bulgaria’s health care system is based on a social health insurance; in 1999 the National Health Insurance Fund was established. Overall, the health care system is described as a public-private mix. Compared to the other Central and Eastern European countries (CEECs), the reforms in health care were initiated only in 1999 and led to some structural changes.  The share of private funding of total health expenditure (42%) and of total pharmaceutical expenditure (even 82%) is high, even in comparison to other countries of the region. One major challenge in health care was a high level of informal payments.  To prepare for its accession to the European Union (EU) in 2007, the ‘acquis communitaire’ in pharmaceutical policy was adjusted. As a result, relevant laws such as the Law on Pharmaceutical Products in Human Medicine were amended several times. New committees were established to support and implement the pricing and reimbursement policy of the government.  In the year of the survey, a slight decrease in the number of medicines on the market was observed, and according to country experts could in the strategy of pharmaceutical companies to withdraw medicines from the market due to possible restrictive pricing procedures or unattractive market conditions. No official National Drug Policy Paper had been adopted at the time of the survey. This was criticized as ‘this leads to the lack of middle and long term vision and sustainability of the development of the pharmaceutical sector and even worse, it gives the possibility each new leading team in the Ministry of Health constantly to make changes in the pharmaceutical environment’.  Despite fewer medicines available on the market, pharmaceutical sales were growing, with an increase of 71% from 2005 till 2009. The relative increase was higher in the out-patient sector than in the in-patient sector which was attributed to restricted hospital budgets, whereas the National Insurance Fund resources for out-patient medicines as well the Ministry of Health budget for public procurement had been slightly extended.  Pharmaceutical consumption increased as well, both in terms of value (prices) and volume. High-cost medicines were included in the reimbursement list of the social health insurance. Medicines for out-patient use in the positive list (introduced in 2003) were, at least partially, funded by the National Health Insurance Fund: medicines for the treatment of chronic diseases which led to several disturbance of the quality of life or disability and required prolonged treatment were reimbursed up to 100 per cent; medicines for chronic diseases occurring with high prevalence were reimbursed up to 75 per cent and other reimbursable medicines were reimbursed up to 50 per cent.  At the time of the survey, the implementation of a comprehensive generics policy had been discussed for many years. Generic substitution was not allowed, and prescribing by International Non-Proprietary Name (INN) was allowed but on a voluntary basis, and it was rarely applied. Apart from the reference price system that included all reimbursed medicines, there were not incentives for patients to ask for less expensive medicines such as generics. Instruments to enhance a more rational use of medicines, including a more rational prescribing, were limited.  Experts in Bulgaria identified as major challenges for pharmaceutical policies: to implement a system to monitor pharmaceutical consumption, prices and expenditure; to improve cooperation between the competent authorities, to improve knowledge and capacity of health professionals and to encourage a more rational use of medicines. | **Overview:**  The Czech Republic was founded in 1993 after the split from Slovakia. Changes in the health care system, to move away from the Semashko model, had already been initiated after the political changes in 1989. In 1993 a statutory social health insurance system was introduced which was initially based on competition of the sickness funds. As a result, several sickness funds went bankrupt; their number was reduced from 27 in 1994 to 14 in 1996. At the end of the 1990-ties there were nine sickness funds; this number was kept stable in the new millennium, including the year of the survey.  Major elements of the early 1990-ties reform were liberalisation and decentralization. A primary health care system, with some kind of gatekeeping, was introduced. From 2003 on hospitals ownership was shifted from state to non-state entities. In 2004 a DRG system for hospital funding was introduced. Pharmaceutical industry, wholesale and pharmacies were privatized, and the number of pharmacies increased. At the time of the survey, pharmacy chains were in place, and wholesalers had purchased pharmacies. Some OTC medicines could be purchased outside pharmacies; dispensing doctors were not permitted.  In 2008 there was a major institutional reform related to medicines. The competence for pricing was transferred from the Ministry of Finance to the Medicines Agency (Státni ùstav pro Kontrolu Léčiv, SUKL), and the competence for reimbursement was transferred from the Ministry of Health to SUKL. SUK became the key actor in the regulation of medicines. This could be seen as a reaction to criticism about a weak regulatory system and an intransparent reimbursement system, with no clear criteria and procedures for reimbursement  In the years before the survey, in pharmaceutical expenditure has been rising which was attributable to an ageing population as well as new and high cost therapies. Discussions about sustainability in health care funding, and the need for changes, had been ongoing for several years.  At the beginning of 2008, co-payments, so-called regulatory fees, were introduced to restrict visits to doctors (CZK 30 / € 1.10 in 2008 for a regular visit, and CZK 90 / € 3.35 for an emergency visit) which led to a decrease in patients’ visits to out-patient specialists by 28% compared to 2007. These ‘regulatory fees’ were also introduced for inpatients’ stay (60 CZK / € 2.24 per day). Since their implementation these co-payments had heavily criticized by, among others, the patients, and there were calls for them to be abolished or reduced.  In the out-patient sector, there are co-payments on medicines. No fixed co-payments resulting from the difference between the pharmacy retail price and the reimbursement price were applicable in the year of the survey, which resulted from the design of the reference price system introduced in 1995. Doctors are required to inform the patient whether the prescribed medicine was fully, partially or non-reimbursable. In addition, a prescription fee of CZK 30 / € 1.10 was introduced in 2008. There are no exemptions for vulnerable groups.  In the year of the survey, the value-added tax on medicines was increased from 5% to 9% which impacted medicine prices and was thus of relevance for the payers (either public payers or patients).  In December 2008 the Czech Republic began to operate a central electronic prescription system that was expected to improve communication between doctors, pharmacies and patients and to enhance rational use of medicines.  The Czech Republic had a rather high pharmaceutical consumption that had been attributed to a high rate of patients’ visits to doctors. The probability that a patient leaving the doctor’s practice had received a prescription was indicated with more than 95 per cent. Pharmaceutical budgets for prescribing doctors were in place in order to rationalize prescribing behaviour. However, sanctions in case of over-prescribing had never been enforced over the years.  Generics uptake in the Czech Republic was high. There was a long tradition of generic manufacturing companies in the company, and instruments such as the reference price system and generic substitution incentivized the use of generics**.** |
| **Key challenges:**   - Underfunding of the pharmaceutical system - High co-payments and out-of-pocket payments in health care, particularly for medicines - Informal payments in health care - High value added tax rate on medicines (20%, same as standard rate) - Availability limitations due to withdrawal of medicines from the market by the pharmaceutical industry - Lack of coordinated pharmaceutical policy - Room for improvement in the pharmaceutical pricing and reimbursement process - Limited enforcement of demand-side measures to promote a more rational prescribing and use of medicines | **Key challenges:**   - Sustainability of funding in health care - High co-payments and out-of-pocket payments in health care, particularly for medicines - High pharmaceutical consumption rate, and expectations by patients on doctors to prescribe a medicine - Limited enforcement of prescribing monitoring |

| **Hungary (2009)** | **Latvia (2008)** |
| --- | --- |
| **Overview:**  In Hungary health care was organised on the basis of a social health insurance, with the National Health Fund (Országos Egészségbiztosítási Pénztár, OEP) playing an important role in the organisation and financing of the health care system. In the family doctor system doctors had contracts with the regional branches of OEP, and they acted as gatekeepers to specialists.  Pharmaceutical spending had posed a major challenge in the years before the survey (2009). Hungary spent one third of its health expenditure on medicines. In 2005, there was overspending of the planned pharmaceutical budget by nearly a quarter. As a result, the Hungarian government decided on a reform whose major goal was to contain public pharmaceutical expenditure. The changes entered into force on 1 January 2007.  This reform changed the pharmaceutical system from 2007 on. It included supply-side measures such as a statutory claw-back for industry and price-volume agreements and extensive price cuts in 2007. Furthermore, in the out-patient sector, the percentage reimbursement rates were decreased, and a prescription fee of HUF 300 (around € 1,-) was introduced. It was argued that affordability was not negatively impacted despite of the massive increase in out-of-pocket payments for patients, as percentage co-payments did not increase, or only increased slightly, in absolute figures due to the price cuts. After the reforms of 2007 public pharmaceutical expenditure decreased considerably.  Hungary’s accession to the EU in 2004 had a major impact on the pharmaceutical pricing and reimbursement process. Before 2004, no systematic and elaborated process for deciding which services should be included or excluded from the statutory benefits package existed, and final decisions were made in a non-transparent way. EU regulation, particularly the Transparency Directive, contributed to strengthening the process and to the introduction of clearer and more transparent criteria. Health Technology Assessment (HTA) increasingly played a role in the appraisal process.  Another element of the 2007 reform was the liberalisation in the pharmacy sector, with the fall of the restrictions on ownership and establishment of pharmacies and the permission of the sale of defined Over-the-Counter (OTC) medicines outside pharmacies. Some of the liberalisation changes were recalled later (after the survey).  The 2007 reform also aimed to increasing generic uptake. Further competitive elements (e.g. more frequent bids by suppliers) were built in the reference price system that had been in place since the 1991. In general, generic uptake had been high in Hungary already. Since the 2007 reform, pharmacists were obliged to inform the patient about cheaper alternatives and to substitute for lower priced medicines unless the doctor or the patient opposed to it. The process was supported by an improved IT support system. Another new measure related to generics was the introduction of a ‘generic price link’ policy resulting in price cuts for generics compared for the previously reimbursed alternative medicines. | **Overview:**  Latvia established a social health insurance in 1998 that was predominantly funded by the central government budget (general taxation). At the time of the survey, a process of decentralisation and reorganisation in health care was on-going. General practitioners (GP) practices were planned to become the basic unit of health care instead of health care centres or policlinics. GPs already had a key role as gatekeepers in referring patients to secondary and tertiary care. Following the Implementation Plan of the Development Programme of Out-patient and In-patient Healthcare Service Providers for 2005-2010, the number of in-patient health care providers was reduced, and these health care providers were empowered to provide day care, out-patient health care and home care.  Latvia had a well-developed pharmaceutical pricing and reimbursement system at a time of the survey, with clear rules and procedures for the medicines to be included and maintained in the publicly funded reimbursement system. Pharmacoeconomics played an important role in the reimbursement process, and the Baltic Guideline for Economic Evaluation of Pharmaceuticals, developed in cooperation with the two other Baltic countries, was included in the regulatory framework. Methodological questions on how to assess the cost-effectiveness of new medicines with limited therapeutic effectiveness were a major challenge for change, though.  Several initiatives had been launched to promote a more rational use of medicines, including a more rational prescribing. Prescribing should be guided by rational pharmacotherapy guidelines that had been developed for several indications. The prescribing pattern was being monitored, with financial penalties foreseen for doctors in case of irrational prescribing. In case of a justification, doctors could apply for an increase in the pharmaceutical prescribing budget target. However, there were cases of irrational use, including irrational marketing, of medicines, which the government planned to address. Generic promotion, with encouraging doctors to prescribe by INN, permitting generic substitution and a reimbursement system (e.g. a reference price system) that incentivized patients to use generics, was part of Latvia’s policies to promote a more rational use of medicines.  At the same time, Latvia faced major problems in the pharmaceutical system. There had been a continuous growth in pharmaceutical expenditure, and limited resources to cover the growth, particularly in the light of the new expensive medicines. Pharmaceutical expenditure per capita was by 25% lower than the CEE-10 average. It had grown by 66% within three years. The share of public funding on medicines was comparatively low, which required high co-payments from the patients.  In addition, Latvia was suffering, like other small markets, from overpaying for medicines (high prices) causing affordability and equity problems, and from limited availability of medicines.  Latvia, together with the Baltic countries, was among the first countries of Europe to be strongly hit by the global financial crisis from 2008 on. In response, Latvia implemented some cost-containment measures shortly after the EHIS survey (end of 2008): At the beginning of 2009, Latvia increased the value-added tax: the standard one (from 18% to 21%) as well as the one on medicines (from 5% to 10%). In March 2009, the percentage reimbursement rates were reduced from 90% to 75% and from 75% to 50%. Further measures (wholesale margin cuts, further increase in co-payments) followed in 2010 and 2011. |
| **Key challenges:**   - High co-payments and out-of-pocket payments for medicines following a radical reform in 2007 to contain public pharmaceutical expenditure - Informal payments in health care - Overpaying the prices of generic medicines - Weak incentives at the demand-side to promote generic uptake (e.g. INN prescribing is not very common in practice) - Limited enforcement of rational use of medicines, including cost-effective prescribing by doctors | **Key challenges:**   - Under-funding of the pharmaceutical system, and growth in pharmaceutical expenditure (aggravated by the global financial crisis), need for budget control and cost-containment - High share of private funding for medicines, and informal payments in health care - Overpaying the prices of medicines - Availability problems as a small market, and regional disparities related to the provision of pharmacies - Irrational use of medicines, including irrational prescribing and irrational marketing |

| **Poland (2009)** | **Romania (2008)** |
| --- | --- |
| **Overview:**  The Polish health care system has been organised as a Social Health Insurance system. In 1998 the National Health Fund (Narodowy Fundusz Zdrowia, NFZ) and its 16 regional sickness funds were established. Public funding has been generated through social health insurance contributions.  Pharmaceutical expenditure slightly increased in the years before the EHIS survey. Poland had a high share of private spending in pharmaceutical expenditure (61%). Around 40% of the medicines authorized were included in the reimbursement list, thus partially or fully funded.  Patients had to co-pay for defined medicines in the out-patient sector: There was a prescription fee for certain indications (so-called basic medicines, e.g. antibiotics), and percentage co-payments of 30% (Parkinson’s disease, Alzheimer’s disease) and 50% (e.g. medicines for menopause, cardiovascular disorders, hypertension). Specific patient groups (e.g. cancer patients, patients with epilepsy and war veterans) were exempt from any kind of co-payment. In the in-patient sector no co-payments were required from patients.  At the time of the survey, a health care reform was still ongoing. In particular, a new Pharmaceutical Law was drafted. It was scheduled to be passed at the end of 2009, but, in fact, it was implemented only from 2012 on. It fundamentally changed the pricing and reimbursement process in Poland. The reform of the pharmaceutical system was also a response to an infringement procedure that the European Commission had launched in December 2005 against Poland for the breach of the EU Transparency Directive. A major concern of the EU was the long time periods till pricing and reimbursement decisions were taken. Furthermore, the Polish pharmaceutical pricing and reimbursement system was considered as not sufficiently transparent, and the rules and procedures of reimbursement process were not in line with the EU Transparency Directive. At the time of the survey, Poland had been working on clearer and more transparent reimbursement criteria, in preparation of the new law, supported by expertise from other EU Member States via a EU Twinning Project. It was recommended that Health Technology Assessment (HTA) and evidence-base medicine should be better considered and the Polish HTA Agency should have a stronger and more independent role.  Another weak element in the Polish pharmaceutical system was limited enforcement and transparency for patients. Doctors were required to prescribe by therapeutic guidelines, but real prescribing behaviour was found not to relate to the guidelines. Generic uptake was promoted by a fast track approval procedure that appeared to be efficient and by demand-side measures such as generic substitution and INN prescribing ,both indicative, not mandatory. Incentives for doctors to prescribe by INN, for pharmacists to dispense generics and for patients to ask for generics were missing. Patients were often not aware that they could ask for a less expensive generic and reduce their co-payments, and they frequently were still reluctant to use generics. | **Overview:**  In 1998 Romania established a statutory social health insurance system. It was organised decentralized under the umbrella of the National Health Insurance Fund (Casei Nationale de Asigurari de Sanatate, CNAS). With the introduction of the social health insurance system, regional health administrations, that had an important role before, lost relevance in terms of organisational competences and financial power.  Given massive under-funding, a major challenge for the Romanian health care system was to ensure the quality of health care providers and services that was considered as inappropriate and not state-of-the-art. Salaries for health care professionals were low. There was still a strong focus on the in-patient sector, and medical services which would be provided in the out-patient setting in other countries were offered in hospitals in Romania. Furthermore, equity was another concern due to strong disparities in health care provision between urban centres and the country-side. Related to access to medicines, great differences in the number of pharmacies in urban and rural areas existed.  In 2008, total pharmaceutical expenditure per capita was nearly 50% lower than the average of the CEE region. However, it had been considerably growing in the years before the survey, and it had increased 2.5 fold since 2003. 55% of pharmaceutical expenditure was funded by private households.  Literature indicated large-scale existence of ‘informal payments’ in the Romanian health care system. Official co-payments related to medicines resulted from the percentage co-payments that were either 10% or 50% of the medicine price in the out-patient sector. 100% coverage for out-patient medicines was granted for specific, often chronic, serious diseases (e.g. diabetes), for medication as part of national health programmes (e.g. cancer medicines) or specific medicines for children, students and pregnant women. Further exemptions from co-payments existed for war veterans and disabled people on low incomes.  Private health insurance, introduced to address the high ‘informal payments’, did not play a role and did not cover co-payments for medicines.  In 2007, one third of the authorized medicines were not available on the market. From 2007 on, Romania applied the so-called ‘sunset clause’, stating that a medicine might lose its market authorization if not marketed within three years. Romania that joined the EU in 2008 implemented EU law related to marketing authorization of medicines into national law in 2006.  Though a high percentage of health care expenditure was spent on the in-patient sector, there was still under-funding of all hospitals that impacted the quality of care. Shortages of medicines and medical devices in hospitals as a result from underfunding were reported.  In the out-patient sector, major accessibility problems resulted from the pharmaceutical budgets defined in the contracts between community pharmacies and the national health insurance. In case that the threshold had been reached within a month, pharmacies were no longer allowed to fill prescriptions and dispense medicines at the expense of the sickness funds (with a few exemptions for diabetes medicines and medicines for a few chronic diseases). The patients were required to purchase their medication out-of-pocket in such a case. This system was heavily criticized and was abolished at the beginning of 2009 (after the EHIS survey). At that time of the survey, following World Bank consultancy several other reforms aimed at making the pharmaceutical pricing and reimbursement system more transparent were planned but then not implemented as planned. Pharmacoeconomics in the reimbursement process played no major role at the time of the survey.  Though there were no pharmaceutical budgets for doctors, there was a monthly prescribing limit corresponding to a value of RON 300 / around € 70 for medicines with a co-payment of 50%. To ensure a more rational prescribing, Romania had prescription guidelines and a prescription monitoring system. However, in practice feedback on prescribing behaviour was reported months or even years later. Enforcement of physician monitoring would be required. Also, though the generic market shares were high, generic uptake could be increasing by making INN prescribing and generic substitution mandatory. |
| **Key challenges:**   - High co-payments and out-of-pocket payments in health care, particularly for medicines; including informal payments in health care - Intransparent pharmaceutical pricing and reimbursement system, with unclear rules, long decision times - Limited enforcement of demand-side measures to promote a more rational use, including prescribing, of medicines | **Key challenges:**   - Massive under-funding of the health care, including pharmaceutical system, impacting negatively quality of care and accessibility - High co-payments and out-of-pocket payments in health care, particularly for medicines; including informal payments in health care - Limited availability of medicines on the market in general, and in pharmacies and hospitals due to underfunding and pharmaceutical budgets for community pharmacies - Insufficient mechanisms to protect vulnerable groups - Intransparent and unclear pharmaceutical pricing and reimbursement procedures - Limited enforcement of demand-side measures to promote a more rational use, insufficient prescription monitoring |

| **Slovenia (2007)** | **Slovakia (2009)** |
| --- | --- |
| **Overview:**  The Slovenian health care system is based on a social health insurance system that was implemented in 1992. Funded by social health insurance contributions, it provided health care for its around 2 million inhabitants. In addition, voluntary health insurance played a major role.  Slovenia’s economic performance considerably better than those of other countries in the region and rather close in the EU average, In 2008, total pharmaceutical expenditure was more than 50% higher than the CEE-10 average, and at the same time the share of health expenditure spent on medicines was declining: amounting to less than 20%, it was closer to shares in Western European countries than to countries in the region. The share of private funding in pharmaceutical expenditure was by a quarter lower than the CEE-10 average whereas the respective share in health expenditure was similar to the other countries in the region.  Co-payments were, in general, not a major issue for the Slovenian population. There were no co-payments in the in-patient sector, and exemptions were granted from co-payments in the out-patient sector for specific social groups and patients with specific diseases (e.g. HIV/AIDS, diabetes). Percentage co-payments of 25% which were charged for several out-patient medicines were usually covered by the voluntary health insurance which around 90% of the population had concluded.  Slovenia adapted its pharmaceutical system to the EU provisions before its accession to the EU in 2004. In the years to follow, further policy measures were taken: In 2006 and 2007, the rules for pharmaceutical pricing were changed, with the aim of increasing transparency and simplification of the procedure, ensuring cost-containment and financial sustainability and including elements of reward for innovation. The changes, which led to price increases for more than 600 medicines as well as price decreases for around 750 medicines, were expected to result in substantial savings for public payers that could invest these financial resources to fund new medicines. New high-priced medicines (particularly cancer medicines were included in the reimbursement list at that time.  Slovenia had a rather high ratio of inhabitants per pharmacy (around 7,400) in comparison to other countries of the region and most European countries. At the same, the pharmacy density should be interpreted considering the topology of the small country.  Slovenia had a comparably high generic market share, and generics appeared to be rather well accepted. This might be attributed to be a strong role of local generic companies as well to appropriate demand-side measures such as generic substitution and INN prescribing. Doctors were obliged to inform patients about co-payments.  A major challenge for Slovenia has been the availability of medicines since the small market might be considered as not sufficiently attractive for industry to supply. Slovenia had fewer than 3,000 presentations (counted by different pharmaceutical forms, dosages and pack sizes) on the market. In the years after the survey, Slovenia was a driver among the EU Member States with a small market to bring this topic on the agenda and to explore policy options. | **Overview:**  Health care is based on a social insurance model. Mandatory health insurance was introduced in 1994; citizens could choose among 12 health insurance institutions. Their number deceased over the years: due to the competitive character in the health insurance market, some health insurance institutions had to close, and others merged. At the time of the survey, there were six health insurance funds. The two biggest ones were considered as statutory, and their solvency was ensured by the State. The health insurance institutions were responsible for funding medicines in the out-patient and the in-patient sectors. While the scope of services covered by the social health insurance was legally defined as rather broad in law, funding was limited. This contributed to high debts of social health insurance to health providers (particularly for the sector of specialised hospitals, but also to pharmaceutical companies and pharmacies).  In the years after Slovakia’s accession to the EU, several pharmaceutical policy reforms had been performed. These included the introduction of a new reimbursement process (e.g. institutional changes such as a composition of an advisory body, shift in the competence for pricing from the Ministry of Finance to the Ministry of Health), the encouragement of price competition for interchangeable medicines and a stronger focus on evidence-base and evaluation of medicines during the reimbursement process. Co-payments were either totally abolished (the fees for out-patient visits and for hospital stays) or reduced (i.e. the prescription fee for medicines of € 0.4 per prescription was decreased to € 0.15) in October 2006. Co-payments on medicines were limited by an annual ceiling. In 2007, around 39% of the medicines on the reimbursement list were either fully reimbursed or had a percentage co- payment which was at maximum € 0.3.  Still, in the years before the survey, private spending shares of health and pharmaceutical shares had risen. Total pharmaceutical expenditure was 50% higher than the CEE-10 average, and it was continuously growing. The VAT rate on medicines used to be quite high, amounting to the standard VAT rate of 19%, but from beginning of 2007 it was reduced to 10%.  These developments took place against the background of substantial economic growth in general and a growth in the pharmaceutical market in Slovakia in the first years of the new millennium. At the time of the survey, Slovakia’s economic growth had started to come to an end, and challenges on how to fund, particularly high-cost, medicines were high on the agenda. For instance, health insurance institutions tendered centrally for high-cost medicines in hospitals. Legislative changes to allow managed entry agreements (i.e. agreements with industry to grant quicker access in return for financial contributions), for instance, were explored. A more rational selection of medicines to be included into reimbursement was planned since the system was considered as no longer reimbursable. Pharmaceutical sales had been growing in volume and value in the years before the EHIS survey. While the increase in value used to be 2 digit 3 years before the survey, it came down to less than 1 per cent.  The number of medicines authorized and on the market in Slovakia was high (around 19,000 presentations), and it had risen in the years before the survey. Around 5,000 medicines (different dosages, pharmaceutical form and pack sizes counted) were included into reimbursement.  Slovakia had comparably high generic market shares, still stable over the years (half of all sales in pharmacy), due to a tradition of generic medicines use (including a historically strong local industry) and to government initiatives to promote generic uptake, e.g. through information activities about generics addressed to the public and INN prescribing and generic substitution. There had been a trend, especially in lower income groups, of pressuring doctors to prescribe equivalent generics with no or lower co-payment, in terms of generic substitution. |
| **Key challenges:**   - Different challenges than most countries of the region but rather similar challenges as Western European countries (e.g. funding new high-cost medicines) - Availability concerns (limited number of medicines on the market) due to its status as a ‘small market’ | **Key challenges:**   - Underfunding of the system challenges a, in principle, generous coverage system, thus limiting access for patients and pressuring market players who did not get paid, or with delays - Global financial crisis hit Slovakia hard and led to cost-containment measures - Funding for new, high-cost medicines is limited - Incentives for generic substitution are considered as weak (for patients) and misaligned (for pharmacies) |

Sources: All: [49]; Bulgaria: [101, 118, 83, 58, 10, 7]; Czech Republic: [113, 10, 7, 9, 109, 110, 82, 100, 120, 121]; Hungary: [104, 85, 64, 10, 12, 106, 21, 16, 121, 91, 119]; Latvia: [102, 107, 114, 86, 88, 122, 60]; Poland: [111, 103, 115, 123, 96, 95, 8, 124]; Romania: [63, 84, 10, 9, 89, 97, 71, 92, 98, 94, 125]; Slovenia: [10, 7, 9, 81, 126, 70, 127]; Slovakia: [41, 105, 108, 128, 85, 100, 116, 129, 116]
